# LEAFY and WAPO1 jointly regulate spikelet number per spike and floret development in wheat

**DOI:** 10.1242/dev.202803

**Published:** 2024-07-31

**Authors:** Francine Paraiso, Huiqiong Lin, Chengxia Li, Daniel P. Woods, Tianyu Lan, Connor Tumelty, Juan M. Debernardi, Anna Joe, Jorge Dubcovsky

**Affiliations:** ^1^ Department of Plant Sciences, University of California, Davis, CA 95616, USA; ^2^ Howard Hughes Medical Institute, Chevy Chase, MD 20815, USA; ^3^ Institute for Plant Genetics, Heinrich Heine University, Düsseldorf 40225, Germany

**Keywords:** Wheat, Inflorescence development, Spike, LFY, Spatial transcriptomics

## Abstract

In wheat, the transition of the inflorescence meristem to a terminal spikelet (IM→TS) determines the spikelet number per spike (SNS), an important yield component. In this study, we demonstrate that the plant-specific transcription factor LEAFY (LFY) physically and genetically interacts with WHEAT ORTHOLOG OF APO1 (WAPO1) to regulate SNS and floret development. Loss-of-function mutations in either or both genes result in significant and similar reductions in SNS, as a result of a reduction in the rate of spikelet meristem formation per day. SNS is also modulated by significant genetic interactions between *LFY* and the *SQUAMOSA* MADS-box genes *VRN1* and *FUL2*, which promote the IM→TS transition. Single-molecule fluorescence *in situ* hybridization revealed a downregulation of *LFY* and upregulation of the *SQUAMOSA* MADS-box genes in the distal part of the developing spike during the IM→TS transition, supporting their opposite roles in the regulation of SNS in wheat. Concurrently, the overlap of *LFY* and *WAPO1* transcription domains in the developing spikelets contributes to normal floret development. Understanding the genetic network regulating SNS is a necessary first step to engineer this important agronomic trait.

## Teaser

**Summary:** The plant-specific transcription factor *LEAFY* plays an important role in the regulation of the number of spikelets per spike in wheat.

## INTRODUCTION

Every year, trillions of wheat spikes mature worldwide carrying the grains that provide one-fifth of the calories and proteins consumed by the human population [according to the Food and Agriculture Organization (FAO) of the United Nations; http://www.fao.org/faostat/en/#data]. Therefore, increasing the maximum number of grains that can be produced by each spike could contribute to remedying the global needs for increased wheat productivity to feed a growing human population.

Wheat spikes, as other grass inflorescences, comprise specialized reproductive organs called spikelets, which are short indeterminate branches. Each spikelet has two proximal sterile bracts (glumes) followed by a variable number of florets. Individual florets include a lemma, which is also a bract, subtending the floral organs (one palea, two lodicules, three stamens and a pistil) (Preston et al., 2009; Debernardi et al., 2020a). The wheat inflorescence meristem (IM) produces multiple lateral spikelet meristems (SMs) in a distichous order before transitioning to a terminal spikelet (henceforth, IM→TS). The timing of this transition and the rate at which the SMs are formed determine the spikelet number per spike (SNS) and the maximum number of grains that can be formed in the spike.

The number of spikelets in a wheat spike is affected by multiple environmental conditions, including drought, salt stress, heat, and reduced nutrients, all of which result in reduced SNS (Frank and Bauer, 1982; Frank et al., 1987; Maas and Grieve, 1990). However, differences in SNS also have a strong genetic component, with broad sense heritability ranging from *H*^2^=0.84 in irrigated fields to *H*^2^=0.59 in water-stressed environments (Zhang et al., 2018). This high heritability has facilitated the identification of several wheat genes involved in the regulation of SNS. *VERNALIZATION1* (*VRN1*), *FRUITFULL2* (*FUL2*) and *FUL3*, the wheat homologs of the *Arabidopsis SQUAMOSA* MADS-box genes *APETALA1* (*AP1*), *CAULIFLOWER* (*CAL*) and *FUL*, have been shown to be essential for spikelet development and for regulation of the IM→TS transition (Li et al., 2019). Loss-of-function mutations in *vrn1* or *ful2* result in normal plants with significant increases in SNS. However, in the *vrn1 ful2* combined mutant the IM remains indeterminate and lateral spikelets are converted into tiller-like organs with vestigial floral organs. These vestigial floral organs disappear in the *vrn1 ful2 ful3* higher-order mutant, in which spikelets revert to vegetative tillers subtended by leaves (Li et al., 2019)*.*

Genes that regulate *VRN1* expression have been shown to affect SNS. *FT1*, the wheat homolog of the *Arabidopsis* florigen *FLOWERING LOCUS T* (*FT*), binds directly to the *VRN1* promoter as part of a floral activation complex, and functions as a transcriptional activator (Li and Dubcovsky, 2008; Li et al., 2015). Mutants (or knockdown transgenic plants) of *FT1* (Lv et al., 2014) or its closest paralog *FT2* (Shaw et al., 2019) show reduced or delayed expression of *VRN1*, which is associated with significant increases in SNS. In contrast, overexpression of these genes results in a precocious IM→TS transition and spikes with very few spikelets (Lv et al., 2014; Shaw et al., 2019). Mutations in *PPD1* that reduce or delay *FT1* expression result in SNS increases (Shaw et al., 2013), whereas mutations in *ELF3* that result in the upregulation of *FT1* and *VRN1* expression reduce SNS (Alvarez et al., 2016). *bZIPC1* encodes a protein that physically interacts with FT2, and its mutants also show a large decrease in SNS (Glenn et al., 2023).

However, the underpinning mechanism by which the recently cloned gene *WHEAT ORTHOLOG OF APO1* (*WAPO1*) (Kuzay et al., 2019, 2022) regulates SNS has not yet been elucidated. *WAPO1* is orthologous to the *Oryza sativa* (rice) gene *ABERRANT PANICLE ORGANIZATION1* (*APO1*), and to the *Arabidopsis* gene *UNUSUAL FLORAL ORGANS* (*UFO*), which are both involved in floral development (Levin and Meyerowitz, 1995; Ikeda et al., 2007; Rieu et al., 2023a). In addition to floral defects, loss-of-function mutations in *WAPO1* or *APO1* result in significant reductions in SNS in wheat (Kuzay et al., 2022) or in the number of branches in the rice panicle (Ikeda et al., 2005), respectively.

In *Arabidopsis*, UFO physically interacts with the plant-specific transcription factor LEAFY (LFY) (Lee et al., 1997; Chae et al., 2008; Rieu et al., 2023b), and the interaction is conserved between the rice homologs APO1 and APO2 (Ikeda-Kawakatsu et al., 2012). The *Arabidopsis* LFY protein activates the class-A MADS-box genes *AP1* (Parcy et al., 1998; Wagner et al., 1999) and *CAL* (William et al., 2004), which are homologous to the wheat *VRN1* and *FUL2* genes. Because *VRN1*, *FUL2* (Li et al., 2019) and *WAPO1* (Kuzay et al., 2022) are all involved in the regulation of SNS, we investigated the role of *LFY* on wheat spike development.

In this study, we demonstrate that LFY physically interacts with WAPO1 and that plants carrying loss-of-function mutations in either or both genes exhibit similar floral abnormalities and similar reductions in SNS as a result of a reduced rate of SM formation. We also show significant genetic interactions for SNS between *LFY* and the meristem identity gene *VRN1*, which, together with its closest paralog *FUL2*, promote the IM→TS transition. Finally, we use single-molecule fluorescence *in situ* hybridization (smFISH) to visualize the spatiotemporal expression profiles of these genes and other floral genes during spike development. These studies reveal a tenfold increase in the ratio between the *SQUAMOSA* MADS-box genes (*VRN1* and *FUL2*) and *LFY* in the distal part of the spike at the time of the IM→TS transition, supporting the opposing roles of these genes in the regulation of SNS.

## RESULTS

### Induced loss-of-function mutations in *LFY* reduce SNS and alter floral morphology

**Fig. 1.**
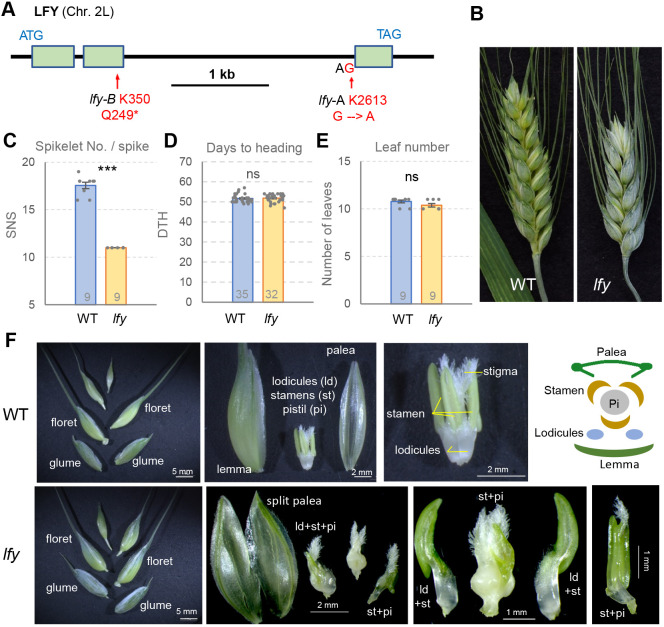
**Characterization of *LFY* loss-of-function mutants.** (A) *LFY* gene structure and selected mutations. (B) Representative spikes of wild-type (WT) and *lfy* mutant plants. (C-E) Comparison between WT and *lfy* for spikelet number per spike (C), days to heading (D) and leaf number (E). Numbers inside bars indicate biological replicates. Dots represent individual plants; error bars represent s.e.m. ***P<0.001. ns, not significant (two-tailed *t-*tests). (F) Top: Images of WT spikelets and schema representing the internal structure. Bottom: Floral abnormalities in *lfy*. ld, lodicule; pi, pistil; st, stamen. See Table S2 for raw data.

Using our sequenced Kronos mutant population (Krasileva et al., 2017), we selected truncation mutations K2613 for *LFY-A* (henceforth *lfy*-*A*) and K350 for *LFY-B* (henceforth *lfy-B*). The *lfy*-*A* mutant has a G>A change in the acceptor splice site of the second intron, which results in mis-splicing of the third exon, a shift in the reading frame, and a premature stop codon that eliminates 121 amino acids (31% of the total protein; Fig. 1A). The *lfy-B* mutant has a premature stop codon at position 249 (Q249*) that truncates 37% of the protein. The eliminated amino acids in the two wheat mutants include the highly conserved LFY DNA-binding domain, suggesting that the truncated proteins can no longer bind their target DNAs and, therefore, are most likely not functional (Maizel et al., 2005; Rieu et al., 2023b) (Fig. 1A). Primers used to track these mutations are described in Table S1. The mutants were backcrossed to Kronos to reduce background mutations, and intercrossed with each other to select sister lines homozygous for the different mutation combinations, including the wild type (WT), *lfy-A*, *lfy-B*, and the *lfy-A lfy-B* combined mutant, designated hereafter as *lfy*.

Comparisons between the homozygous sister lines, raised in a growth chamber, revealed a highly significant decrease (37%, *P<*0.001) in SNS in the combined *lfy* mutant relative to the WT (Fig. 1B,C, Table S2). Smaller but still significant decreases in SNS were detected for the single *lfy-A* (12%) and *lfy-B* (8%) mutants (Fig. S1A, Table S3), which indicates that modification of *LFY* gene dosage can be used to fine-tune SNS in wheat. No significant differences in heading time or leaf number were detected between the combined *lfy* mutant and the WT (Fig. 1D,E, Table S2), suggesting a limited effect of *LFY* on the timing of the transition between the vegetative and reproductive meristems.

In addition to its effects on SNS, *lfy* showed severe alterations in floral organs (Fig. 1F). We quantified the frequency of the defects in 27 first and 27 second florets from spikelets located in the basal, central and distal parts of the spike (Fig. S1B,C, Table S2). The glumes and lemmas developed normally, but 18.5% of the paleas were bifurcated (Fig. 1F, Table S2). Eighty-one percent of the paleas were fused with either lodicules or stamens (Table S2). Lodicules were also fused to stamens or membranous structures. The average number of normal stamens was reduced to 1.4 (Table S2), and one-fifth of the florets showed abnormal stamens and fusions with lodicules, membranous structures or pistils. Only 9% of the florets showed single pistils (primarily those with three normal anthers) and the rest showed more than one pistil and frequent homeotic conversions between stamens and pistils (Fig. 1F, Fig. S1, Table S2).

### Overexpression of *LFY* partially rescues the reduced SNS phenotype of *lfy*

To test whether *LFY* function was sufficient to rescue the mutant phenotypes, we generated transgenic plants expressing the *LFY-A* coding region fused to a C-terminal HA tag and driven by the constitutive maize *UBIQUITIN* promoter. Transgenic lines for the five independent *UBI:LFY-HA* events, all showed significantly higher *LFY* transcript levels in the leaves than did non-transgenic sister lines and WT Kronos, which showed no expression of endogenous *LFY* in this tissue (Fig. S2A, Table S4). Among 14 dissected florets, we observed missing or fused lodicules in 21%, fused stamen filaments in 43% and pistils with extra stigmas in 36% (Fig. S2B,C). Floral organ defects were less frequent and less severe than in *lfy*, which explains the higher fertility of the *UBI:LFY-HA* plants (23±10 grains/plant; mean±s.e.m.) relative to *lfy* (2.7±0.7 grains/plant), but its reduced fertility relative to the WT (94±25 grains/plant, *P*=0.019; Table S5). All errors indicated in the text and figures are s.e.m. These results indicate that the ectopic expression of *LFY* is associated with negative pleiotropic effects on floral organ development and fertility.

**Fig. 2.**
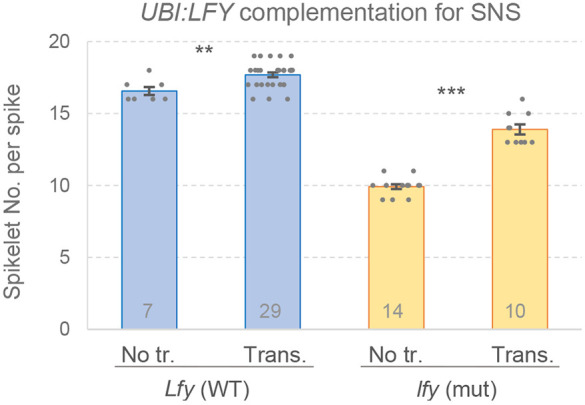
**Effect of *UBI:LFY-HA* on spikelet number per spike (SNS).** Effect of the *UBI:LFY-HA* transgene on SNS in WT and *lfy*. Numbers within bars indicate biological replicates. Dots represent individual plants; error bars represent s.e.m. **P<0.01, ***P<0.001 (two-tailed *t-*tests). ns, not significant. See Table S5 for raw data.

We then crossed the *UBI:LFY-HA* transgenic plant #4 with *lfy*. In the progeny, we selected sister lines homozygous for combined *lfy* mutations or for WT alleles, each with or without the transgenes. Among the plants without the transgene, the combined *lfy* mutants showed reduced SNS (6.6 spikelets, *P<*0.001), as in previous experiments. In the presence of the WT *LFY* alleles, transgenic plants showed 1.1 more spikelets per spike than non-transgenic controls (*P*=0.0045; Fig. 2, Table S5). The effect was larger in *lfy* transgenic plants, which showed four more spikelets per spike than the controls (*P<*0.001; Fig. 2).

### Wheat LFY and WAPO1 show physical and genetic interactions

**Fig. 3.**
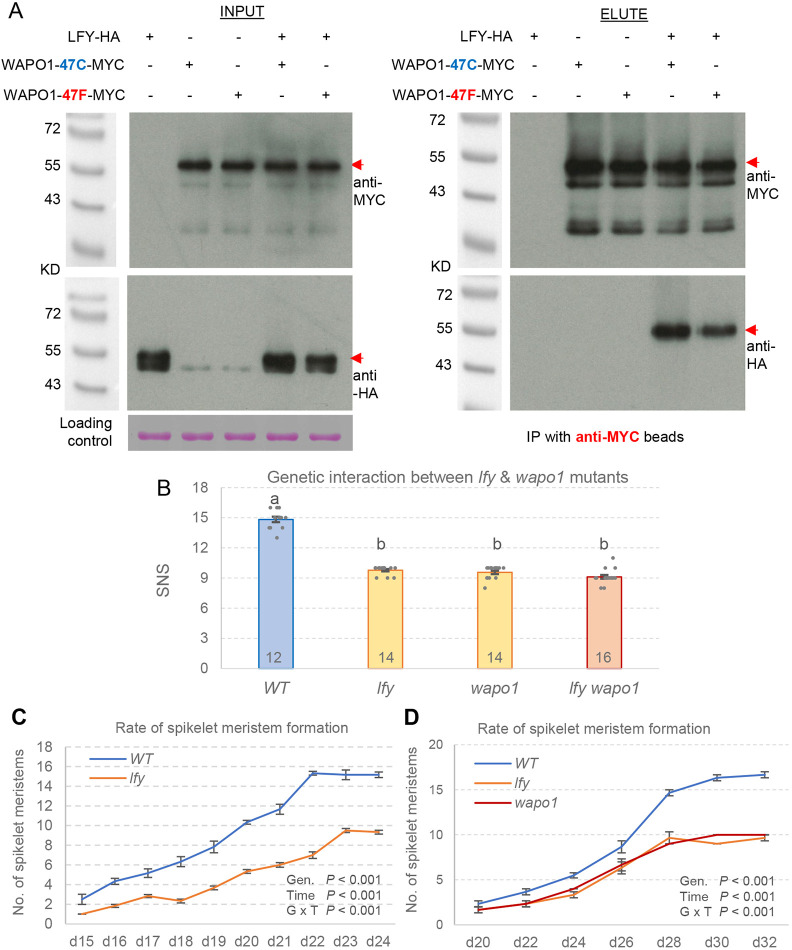
**Physical and genetic interactions between WAPO1 and LFY.** (A) Interaction between LFY and WAPO1 alleles 47F-47C in Kronos leaf protoplasts by co-immunoprecipitation. (B) Genetic interaction between *lfy* and *wapo1*. Different letters indicate significant differences in Tukey tests (*P*<0.05), and numbers inside bars biological replications. Dots represent individual plants; error bars represent s.e.m. (C,D) Changes in the number of SMs with time. d, days from germination. (C) WT and *lfy* mutant grown under long days (*n*=6). (D) WT, and *lfy* and *wapo1* mutants grown for 8 days under short days and then long days (*n*=3). *P-*values correspond to a repeated measures analysis. Error bars are s.e.m. See Table S6 for raw data.

Because *LFY* and *WAPO1* mutants are both associated with similar reductions in SNS (Kuzay et al., 2022), and their homologous proteins interact with each other in *Arabidopsis* (Chae et al., 2008) and rice (Ikeda-Kawakatsu et al., 2012), we tested the ability of LFY and WAPO1 proteins to interact physically with each other in wheat. We used co-immunoprecipitation (Co-IP) to test the interaction between LFY-A and two WAPO-A1 natural alleles that differ in the presence of a cysteine or a phenylalanine at position 47 to determine whether this polymorphism affects the interaction (Kronos carries the 47C allele). The *WAPO-A1-*47F allele was previously associated with higher SNS than the *WAPO-A1-*47C allele (Kuzay et al., 2022). We co-transformed wheat leaf protoplasts with *UBI:LFY-HA* combined with either *UBI:WAPO1-47C-MYC* or *UBI:WAPO1-47F-MYC*. After immunoprecipitation with anti-MYC beads, we detected LFY-HA using an anti-HA antibody in both the WAPO1-47C-MYC and WAPO1-47F-MYC precipitates (Fig. 3A). These results indicate that LFY can interact with both WAPO-A1 variants in wheat.

To investigate whether the physical interaction between LFY and WAPO1 is reflected in a genetic interaction for SNS, we intercrossed *lfy* with a loss-of-function *wapo1* mutant containing early truncation mutations in both *WAPO-A1* and *WAPO-B1* (Kuzay et al., 2022). Owing to the reduced fertility of the homozygous *lfy* mutants (Table S5), we first selected lines homozygous for the *lfy-A* mutant allele and heterozygous for *lfy-B* among F_2_ and F_3_ progenies. We then screened a large F_4_ segregating population and selected the four homozygous classes (WT, *lfy*, *wapo1*, and combined *lfy wapo1*) using molecular markers (primers in Table S1). Plants homozygous for mutations in either *LFY* (*lfy*) or *WAPO1* (*wapo1*) showed large and similar reductions in SNS relative to the WT (34% and 35% reduction, respectively; Fig. 3B). Interestingly, the combined *lfy wapo1* mutant showed a reduction of 38%, relative to the WT, which was not significantly different from the reductions observed in the single mutants (Fig. 3B, Table S6). The genetic epistatic interaction for SNS was highly significant in a factorial ANOVA, and the contrasts for the simple effects showed no significant differences in SNS for *LFY* or *WAPO1* in the presence of the mutant allele of the other gene (Table S6). These results indicate a reciprocal recessive epistatic interaction between these two genes, and that LFY and WAPO1 need each other to regulate SNS.

To determine whether the *lfy* reduction in SNS was due to a premature IM→TS transition or a reduced rate of SM formation, we dissected developing spikes and recorded the variation in SM number per day (sm/d). The first experiment (long days), showed a similar IM→TS transition time but a significantly faster rate of spikelet formation in the WT (1.83 sm/d) than in *lfy* (0.86 sm/d) (Fig. 3C, Table S6). In the second experiment, we grew the seedlings for 8 days under short days and then transferred them to long days to synchronize the reproductive transition. In this experiment, the rate of spikelet meristem formation in the WT (1.40 sm/d) was also faster than in the *lfy* (0.73 sm/d) and *wapo1* (0.83 sm/d) (Fig. 3D). In both experiments, different rates of SM formation were observed from the earliest stages of spike development (Fig. 3C,D). Repeated measures analyses revealed highly significant differences between mutant and WT genotypes, time points, and genotype×time interactions, which indicates a differential response in time (Table S6). There was no significant difference in the rate of SM formation when comparing the *lfy* and *wapo1* mutants alone (Table S6).

### *LFY* and *WAPO1* show dynamic expression profiles during wheat spike development

**Fig. 4.**
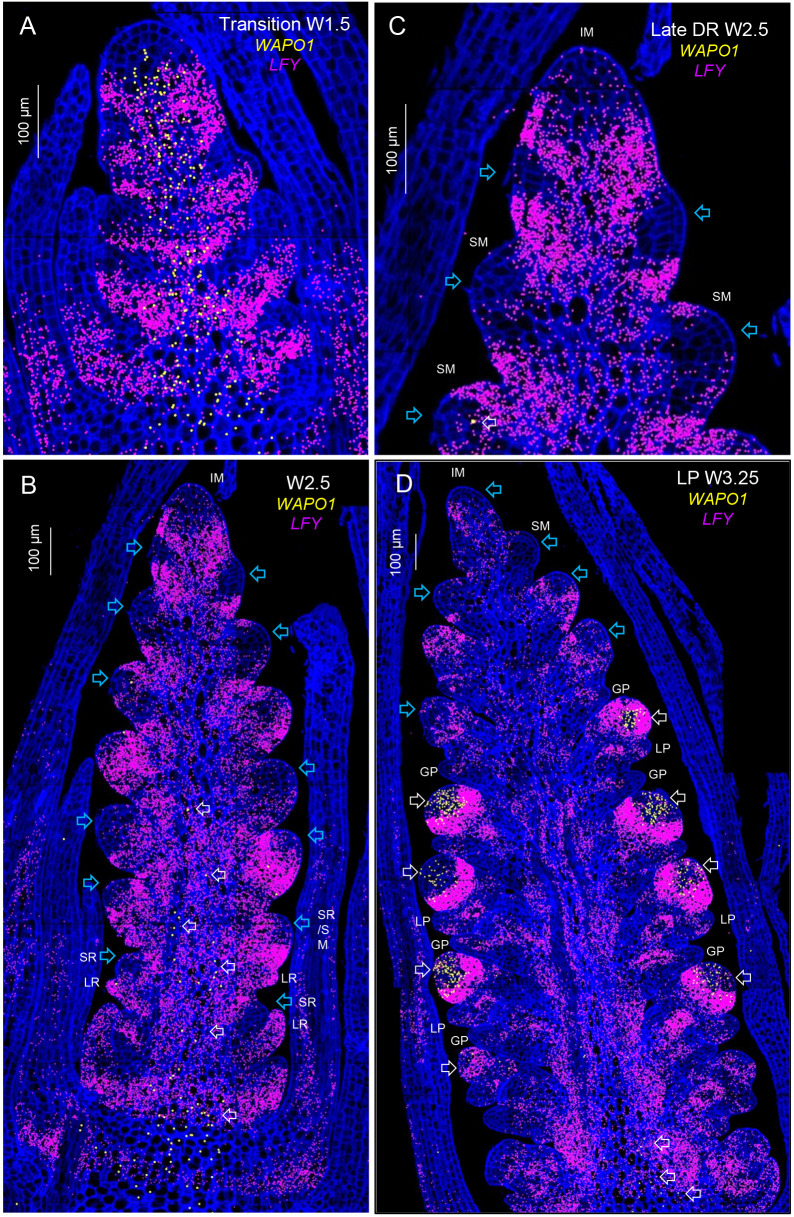
**Single-molecule fluorescence *in-situ* hybridization (smFISH) of *LFY* and *WAPO1* during spike development.** Cell walls stained with calcofluor are presented in dark blue. (A) Elongated shoot apical meristem transitioning from a vegetative to an inflorescence meristem (IM, W1.5). (B) Late double-ridge stage (W2.5). (C) Detail of the IM region from B. (D) Lemma primordia stage (W3.25). Blue arrows indicate regions of the SM where *LFY* expression is lower and white arrows show regions of *WAPO1* expression. GP, glume primordium; LP, lemma primordium; LR, leaf ridge; SR, spikelet ridge. Scale bars: 100 μm. W, Waddington scale (Waddington et al., 1983).

A previous RNA-sequencing (RNAseq) study including different tissues at different developmental stages in Chinese Spring (CS) wheat detected *LFY* transcripts in developing spikes and elongating stems (Choulet et al., 2014) (Fig. S3A). A separate RNAseq study including five spike developmental stages in tetraploid wheat Kronos (VanGessel et al., 2022) demonstrated the presence of transcripts for both *LFY* and *WAPO1* at all five stages. *LFY* transcript levels were more abundant than those of *WAPO1*, and both genes showed lower transcript levels in the apical region at the vegetative stage than at the double-ridge to floret primordia stages (Fig. S3, Table S7). These studies indicate that *LFY* and *WAPO1* are present at the same stages of spike development*.* To refine the localization of *LFY* and *WAPO1* transcripts within the developing spike, we examined their dynamic spatial patterns using smFISH (Fig. 4). For all the smFISH studies, we only compared hybridization signals across developmental stages for individual genes because comparisons of total expression levels among genes are affected by probe sensitivity and can be misleading.

During the transition between the vegetative and reproductive phases (W1.5 in Waddington scale; Waddington et al., 1983), *LFY* transcripts were concentrated in bands radiating from the axis of the elongating shoot apical meristem towards the lateral primordia (Fig. 4A). Only a few *LFY* transcripts were detected at the tip of the IM at this or later spike developmental stages (Fig. 4C,D). At the late double-ridge stage (W2.5), in the less-developed lateral meristems present at the bottom (Fig. 4B) and top (Fig. 4C) of the developing spike, *LFY* expression was stronger at the leaf ridge (also known as the lower ridge) than at the spikelet or upper ridge (Fig. 4B,C, blue arrows). In the more mature SMs located in the central part of the developing spike, *LFY* expression was abundant in the basal region but low in central-distal regions of the SMs (Fig. 4B, blue arrows), suggesting that low *LFY* levels may favor spikelet development. Some SMs showed a more uniform distribution of *LFY*, but those may be the result of off-centered sections.

We used the gene *FRIZZY PANICLE* (*FZP*, *TraesCS2A02G116900*) as an early marker for the IM→TS transition. At W2.5, *FZP* was not detected in the distal part of the wheat spike and was present only at the axils of the developing glumes in the more mature central spikelets, similar to previously reported results in rice (Komatsu et al., 2003). However, at W3.25, when the developing spikes reach the final SNS, *FZP* was detected in the youngest lateral meristems immediately below the IM (Fig. S4, Table S8), indicating their transition to glumes and serving as a marker of the IM→TS transition.

At the W3.25 stage, the more-developed spikelets at the center of the spike showed glume and lemma primordia (Fig. 4D). In these spikelets, *LFY* transcripts were highly expressed within a narrow band that, in a tridimensional space, may appear similar to a bird's nest located distal to the lemma primordia. This high-expression *LFY* band delimited a distal region of the developing spikelet that has lower *LFY* and higher *WAPO1* hybridization signals (Fig. 4D).

*WAPO1* transcripts were detected at the axis of the developing spike at W1.5, in a region that overlapped with *LFY* (Fig. 4A, Fig. S5). However, at later stages (W2.5 and W3.25) *WAPO1* expression was restricted to the base and center of the developing spike, likely in the differentiating vascular tissue (Fig. 4B,D). This distribution is easier to visualize in Fig. S5, which presents *WAPO1* expression alone. At the lemma primordia stage (W3.25), *WAPO1* expression was also detected in the distal part of the more developed spikelets (Fig. 4D, Fig. S5C), in agreement with previous *in situ* hybridization results (Kuzay et al., 2022). A detail of the *WAPO1* expression domain shows colocalization with *LFY* in multiple cells within the distal region of the developing spikelet, which extends to one or two cell layers into the area of high *LFY* expression (Fig. S6).

In summary, the distinct but partially overlapping expression domains of *LFY* and *WAPO1* in the developing spikelets generate an area of overlap, which likely favors interactions between their encoded proteins and provides important spatial information for normal floral development.

### Spatiotemporal expression profiles of *LFY* and *SQUAMOSA* MADS-box genes

In *Arabidopsis*, LFY activates the meristem identity genes *AP1* (Parcy et al., 1998; Wagner et al., 1999) and *CAL* (William et al., 2004), so we first investigated whether the homologous wheat *VRN1* and *FUL2* genes (Table S9) were also regulated by *LFY* using qRT-PCR (Fig. S7, Table S10). We found no significant differences between *lfy* and the WT control for *VRN1* or *FUL2* transcript levels at W2.0, W3.0 or W4.0 (Fig. S7). Analyses of previously published RNAseq data for Kronos spike development (VanGessel et al., 2022) showed that *VRN1* is induced earlier and is expressed at higher levels than the other two *SQUAMOSA* genes, with *FUL2* expressed at higher levels than *FUL3* (Fig. S8A). In the same RNAseq study, *LFY* was expressed at low levels in the vegetative meristem (W1.0) and increased rapidly during W2.0 and W3.0 (Figs S3B, S8A).

**Fig. 5.**
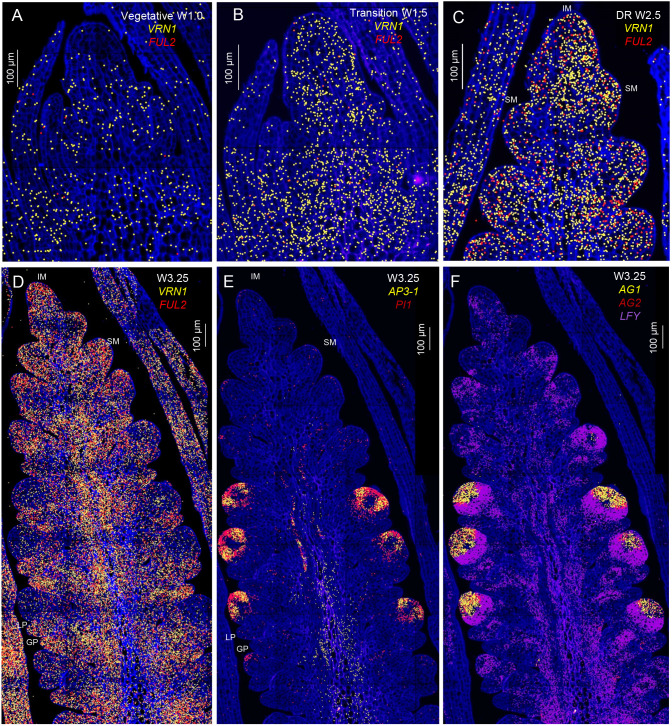
**Transcription profiles of MADS-box floral genes during wheat spike development.** (A-C) Relative distribution of *VRN1* and *FUL2* at late vegetative shoot apical meristem (A), transitioning shoot apical meristem (B) and late double-ridge (C) stages. (D-F) Expression of *VRN1* and *FUL2* (D), the class-B MADS-box genes *AP3-1* and *PI1* (E) and the class-C MADS-box genes *AG1* and *AG2* (F) at lemma primordia stage. DR, double-ridge; GP, glume primordium; IM, inflorescence meristem; LP, lemma primordium; SM, spikelet meristem. Gene identifications and rice orthologs are in Table S9.

We then compared the smFISH spatial and temporal expression profiles of *VRN1* and *FUL2* during spike development. At the late vegetative stage (W1.0), the hybridization signal of *VRN1* was relatively low and *FUL2* was not detected in the apical meristem (Fig. 5A). The signal for both genes increased during the early transition to the reproductive stage, although *FUL2* remained low (Fig. 5B; W1.5). These results were consistent with the RNAseq data (Fig. S8A). At later stages (W2.5 and W3.25), *VRN1* and *FUL2* were both highly expressed in the IM and young lateral SMs (Fig. 5C,D). In the more developed spikelets, located at the center of the developing spike, *VRN1* and *FUL2* expression was stronger at the glume and lemma primordia than in the distal region (Fig. 5D; W3.25), which overlapped with the *WAPO1* expression domain (Fig. 4D). *FUL3* showed a similar spatial expression profile as *FUL2* and it is presented separately (Fig. S9) because of its lower expression levels in the RNAseq data (Fig. S8A) and limited impact on SNS (Li et al., 2019).

To quantify *VRN1*, *FUL2* and *LFY* expression changes in the distal part of the developing spike between the late double-ridge stage (W2.5) and the start of the transition to a terminal spikelet (W3.25), we calculated their signal density (hybridization signal per 100 μm^2^). In both the IM and the IM plus the two youngest lateral meristems (IM+2LM), we observed a three- to fourfold increase in *FUL2* signal density (Fig. S8B) and a 53-69% increase in *VRN1* between W2.5 and W3.25, although only the differences for *FUL2* were significant (Fig. S8C, Table S8). Similar results were obtained when the *VRN1* and *FUL2* hybridization signals were normalized using the *CDC20* signal (Table S8). In the same tissue sections, we detected a 78% decrease in *LFY* signal density (Fig. S8D), and these changes were significant or highly significant depending on the normalization method used (Table S8).

Analyses of the ratios between the *SQUAMOSA* and *LFY* signals showed that the *FUL2/LFY* ratio increased more than 20-fold between W2.5 and W3.25 in both the IM and IM+2LM (Fig. S8E, Table S8). Similarly, *VRN1/LFY* ratios increased eightfold between the same developmental stages (Fig. S8E, Table S8). Because the *SQUAMOSA/LFY* ratios are independent of the normalization method used, and they are determined in the same tissue sections, they provide the best evidence of a significant increase in the expression of the *SQUAMOSA* genes relative to *LFY* in the distal part of the developing spike at the time of the IM→TS transition.

### Spatial expression profiles of floral organ identity genes

We also characterized the spatial distribution of MADS-box genes involved in floral organ development (Table S9). The hybridization signals of class-B (*AP3-1* and *PI1*; Fig. 5E), class-C (*AG1*and *AG2*; Fig. 5F) and class-E (*SEP3-1* and *SEP3-2*; Fig. S10) floral organ identity genes were concentrated in a distal region of the developing spikelets, where they overlapped with the expression of *WAPO1* (Figs 4D and 5D). *SEP1-2*, *SEP1-4* and *SEP1-6* were expressed outside of the region where the two *SEP3* genes were expressed (Fig. S10), suggesting functional divergence between the *SEP1* and *SEP3* genes in wheat.

Finally, we used qRT-PCR to characterize the effect of the *lfy* mutation on the expression of the floral organ identity genes in the wheat developing spike at W4.0, when these genes are highly expressed (Kuzay et al., 2022). The *lfy* mutant showed a significant downregulation of *AP3-1* and *PI1* (Fig. S11A), *AG1* (Fig. S11B), *SEP3-1* and *SEP3-2* (Fig. S11C) relative to the WT (Table S11). Taken together, these results indicate that *LFY* plays an important role in the direct or indirect regulation of the floral organ identity genes.

### Genetic interactions between *LFY* and class-A MADS-box genes

Given the opposite effects of *LFY* and the MADS-box genes *VRN1* and *FUL2* on SNS and their opposite expression changes during the IM→TS transition, we examined their genetic interactions for this trait. We crossed a plant homozygous for *lfy-A* and heterozygous for *lfy-B* with mutants homozygous for *vrn1* and *ful2*-*A* but heterozygous for *ful2-B* and, in the progeny, selected sister plants homozygous for the four gene combinations for each gene (WT, *lfy*, *vrn1*, *lfy vrn1* and WT, *lfy*, *ful2*, *lfy ful2*).

**Fig. 6.**
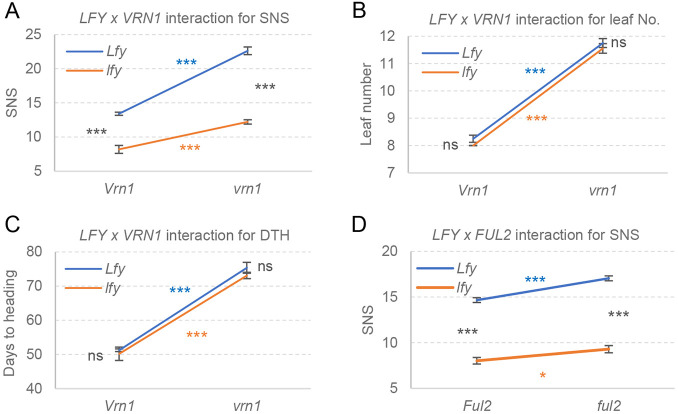
**Genetic interactions between *LFY* and the *SQUAMOSA* MADS-box genes *VRN1* and *FUL*2.** (A-C) Interaction graphs between *LFY* and *VRN1* for SNS (total *n*=34) (A), leaf number (total *n*=34) (B) and days to heading (DTH) (total *n*=34) (C). (D) Interaction between *LFY* and *FUL2* for SNS (total *n*=54)*.* In the interaction graphs, parallel lines indicate additive effects and non-parallel lines reflect interactions. 2×2 factorial ANOVA with *P-*values indicating the four simple effects (Table S12). *P<0.05, **P<0.01, ***P<0.001. ns, not significant. Error bars are s.e.m. See Table S12 for raw data.

A factorial ANOVA including the four homozygous *VRN1-LFY* combinations showed highly significant effects on SNS for both *VRN1* and *LFY*, and a highly significant interaction between these two genes (*P*<0.0001; Fig. 6A, Table S12). The effect of *LFY* on SNS was stronger in the *vrn1* mutant (10.3 spikelets) than in the presence of the functional *Vrn1* allele (5.2 spikelets). In contrast, the effect of *VRN1* on SNS was stronger in the presence of the functional *LFY* allele (9.1 spikelets) than in the presence of the *lfy* combined mutant (4.0 spikelets; Fig. 6A). *VRN1* also showed highly significant effects on the number of leaves and heading time, similar to previous studies (Li et al., 2019), whereas *LFY* showed no significant differences for these traits in the presence of the *Vrn1* or *vrn1* alleles. No significant interactions between these two genes were detected for these two traits (Fig. 6B,C, Table S12).

The effect of *FUL2* on SNS was smaller than the effect of *VRN1* (Fig. 6D; adjusted means from two experiments), but the main effects of both *LFY* and *FUL2* were still highly significant in both experiments. The contrasts for the four single effects were also consistent between the two *FUL2* experiments (Table S12) and also with the interactions between *LFY* and *VRN1*: a stronger effect of *LFY* on SNS in the presence of the mutant *ful2* allele than in the presence of the WT *Ful2* allele, and a stronger effect of *FUL2* in the presence of the WT *LFY* allele than in the presence of *lfy* (Table S12). The *FUL2*×*LFY* interaction was significant only in the second experiment with a higher number of replications (*P*=0.0175). In summary, these interactions suggest the existence of a cross-talk between the *SQUAMOSA* and *LFY* genes in the regulation of SNS.

## DISCUSSION

### Similarities and differences in *LFY* function between *Arabidopsis* and wheat

The most conserved functions of *LFY* across the flowering plants are those associated with the regulation of organ identity in the three inner floral whorls, which include pistils, stamens and petals in eudicot or lodicules in grass species (Yoshida, 2012). *LFY* mutations have more limited effects on bracts (lemmas in grasses) or on the outermost floral whorls, including sepals in *Arabidopsis* and paleas in grasses. However, the first floret of the basal spikelet frequently showed a bifurcated palea (Fig. 1F), suggesting that interactions between *LFY* and genes expressed in the base of the wheat spike regulate palea development.

In the grass species, similar defects in the inner floral organs have been observed for wheat *lfy* mutants (Fig. 1F, Table S2), rice *apo2* mutants (Ikeda-Kawakatsu et al., 2012), barley *multiovary 5* mutants (Selva et al., 2021), and maize *zfl1 zfl2* mutants (Bomblies et al., 2003). These defects include fused organs, reduced number and altered morphology of lodicules (including transformation into bracts and fusions with stamens), reduced number of stamens and increased number of pistils. Fused lodicules with stamens and homeotic conversions of stamens into pistils were observed frequently in the wheat *lfy* mutant (Fig. 1F). The *Arabidopsis* strong *lfy* mutants fail to develop flowers, but weak *lfy* mutants show petals transformed into small sepals or mosaic organs, reduced stamen numbers and increased pistil numbers (Huala and Sussex, 1992; Weigel et al., 1992), similar to the grass species.

Despite the conserved roles of *LFY* in floral organ development, there are also important differences in *LFY* functions between *Arabidopsis* and grasses. First, strong *lfy* mutations in *Arabidopsis* result in the replacement of most flowers by shoots subtended by cauline leaves and the few observed late flowers exhibit intermediate inflorescence characteristics (Schultz and Haughn, 1991; Weigel et al., 1992). In contrast, floret meristems initiate normally in *lfy* in wheat (Fig. 1F) and other grasses (Bomblies et al., 2003; Ikeda-Kawakatsu et al., 2012), and defects appear only later at the inner floral whorls. These results indicate that *LFY* is required to confer the initial floral meristem identity in *Arabidopsis* but not in the grass species.

This difference likely contributes to the opposite functions of *LFY* in inflorescence development in these species. In *Arabidopsis*, constitutive expression of *LFY* (*35S:LFY*) results in conversion of both apical and axillary meristems into terminal flowers, demonstrating that *LFY* is a limiting factor defining when and where flowers are produced (Weigel and Nilsson, 1995). In contrast, constitutive expression of *LFY* in wheat increases the number of lateral spikelets (Fig. 2). Mutations in *LFY* also result in contrasting effects in eudicots and grasses inflorescence development. Weak *lfy* mutants delay the formation of flowers and increase the number of secondary branches in *Arabidopsis* and other eudicot species (Coen et al., 1990; Schultz and Haughn, 1991; Weigel et al., 1992; Souer et al., 1998; Molinero-Rosales et al., 1999). In contrast, *LFY* loss-of-function mutations result in significant reductions in SNS in wheat (Fig. 1B-E), and in the number of branches in rice panicles (Ikeda-Kawakatsu et al., 2012) and maize male inflorescences (Bomblies et al., 2003).

In summary, *LFY* has a similar role on floral organ development in *Arabidopsis* and grasses, but plays different roles in floral meristem identity and inflorescence development.

### LFY and WAPO1 jointly regulate floret and spike development

LFY and WAPO1 proteins physically interact with each other in wheat (Fig. 3A), barley (Selva et al., 2021), rice (Ikeda-Kawakatsu et al., 2012) and *Arabidopsis* (Chae et al., 2008). In *Arabidopsis*, the LFY-UFO complex binds to a different set of genes than LFY alone (Rieu et al., 2023b), including class-B genes *AP3-1* and *PI.* In addition to the class-B genes, *ufo* mutations in other eudicot species show altered expression of class-C genes (snapdragon), class-E genes (cucumber) and both class-C and -E genes (petunia; reviewed by Rieu et al., 2023a). The simultaneous regulation of class-B, -C and -E MADS-box genes seems to be conserved in the temperate grasses. *lfy* mutations in wheat (Fig. S11) and barley (Selva et al., 2021), and *wapo1* mutants in wheat (Kuzay et al., 2022) have all been associated with the downregulation of class-B (*AP3-1* and *PI1*), class-C (*AG1*) and class-E (*SEP3*) floral organ identity genes. This result explains the similar floral defects observed in the wheat *lfy* (Fig. 1F) and *wapo1* mutants (Kuzay et al., 2022), and indicates that both LFY and WAPO1 are required for the proper regulation of these floral organ identity genes.

In addition to their roles in floral development, *LFY* and *WAPO1* jointly regulate SNS. The *wapo1*, *lfy* and combined *lfy wapo1* mutants all show similar reductions in SNS (Fig. 3B). Similar reductions in the number of panicle branches were also observed in the *apo1*, *apo2* and combined *apo1 apo2* mutants in rice (Ikeda-Kawakatsu et al., 2012). In this study, we show that the reduction in wheat SNS in the *lfy* and *wapo1* mutants is the result of a reduction in the rate of SM formation per day rather than a change in the timing of the IM→TS transition. Interestingly, overexpression of either *LFY* (*FALSIFLORA*) or *UFO* (*ANANTHA*) in tomato can transform multiflowered inflorescences into solitary flowers (MacAlister et al., 2012). By contrast, heterochronic shifts during meristem maturation of *UFO* and few other flowering regulators can modulate inflorescence complexity (Lemmon et al., 2016). These results suggest that LFY and UFO homologs can affect meristem maturation rates in both tomato and wheat.

In summary, *LFY* and *WAPO1* act jointly to regulate both inflorescence architecture and floral organ development in wheat, and likely in other plant species.

### Interactions between *LFY* and *SQUAMOSA* genes modulate their opposite effects on the IM development

MADS-box transcription factors act as master regulators of developmental switches and organ specification, with meristem identity genes from the *SQUAMOSA* clade playing essential roles in the initiation of flower development. Among them, AP1 can modulate chromatin accessibility and facilitate access of other transcriptional regulators to their target gene, suggesting that it acts as a pioneer transcription factor (Pajoro et al., 2014). In the wheat *vrn1 ful2 ful3* combined mutant, lateral spikelets are completely transformed into vegetative tillers subtended by leaves (Li et al., 2019), whereas the flowers in the *Arabidopsis ap1 cal ful* triple mutant are transformed into leafy shoots (Ferrándiz et al., 2000). LFY is also a pioneer transcription factor that can bind nucleosomes in closed chromatin, displace H1 linker histones and recruit the SWI/SNF chromatin-remodeling complex, permitting the binding of other transcription factors (Jin et al., 2021; Lai et al., 2021; Yamaguchi, 2021).

In *Arabidopsis*, LFY positively regulates the expression of *AP1* and *CAL* (Parcy et al., 1998; Wagner et al., 1999; William et al., 2004), thereby indirectly regulating their downstream targets. *LFY* also directly regulates hundreds of genes independently of *AP1* and *CAL* (Goslin et al., 2017). However, induction of *LFY* in *ap1 cal* mutants is insufficient to rescue the limited and late formation of flowers observed in this mutant and, instead, results in a lower proportion of plants with flowers than in the control (Goslin et al., 2017). This last result indicates that, in the absence of *AP1* and *CAL*, *LFY* can inhibit flower formation in *Arabidopsis*, similar to its function in the grass species. These results also suggest that the ability of LFY to directly regulate the *SQUAMOSA* genes in *Arabidopsis* but not in wheat (Fig. S7) likely contributes to the opposite functions of *LFY* in the regulation of IM development between these species.

Approximately 200 genes have been identified in *Arabidopsis* as high-confidence direct targets of both LFY and AP1 (Winter et al., 2015). Many of the shared genes directly regulated by the induction of *AP1* or *LFY* show changes in expression with identical directionality, but some of them are regulated in opposite directions, including several key regulators of floral initiation (Goslin et al., 2017). These common gene targets can contribute to the epistatic genetic interactions between *LFY* and *AP1* and to their ability to coordinate the transcriptional programs required for flower initiation and early flower development in *Arabidopsis*.

Genes that are directly regulated by both LFY and SQUAMOSA are likely to also exist in wheat, and may contribute to the significant genetic interaction for SNS observed in this study between *LFY* and both *VRN1* (Fig. 6A) and *FUL2* (Fig. 6D). The net effect of the *LFY*×*VRN1* interaction, 5.2 spikelets per spike, was smaller than the maximum differences of 14.4 spikelets observed between the plants carrying the *vrn1 Lfy* combination (22.6 spikelets/spike) and those carrying the *Vrn1 lfy* combination (8.2 spikelets/spike) (Table S12). These results indicate that the interaction between *LFY* and *SQUAMOSA* explains only part of the variation in SNS.

In summary, *lfy* mutations have opposite effects on SNS than *vrn1* or *ful2* mutations, and those effects are mostly additive. However, significant genetic interactions between *LFY* and the *SQUAMOSA* genes contribute to the observed differences in SNS in the different mutant combinations.

### *LFY* and *WAPO1* show dynamic spatiotemporal expression patterns during wheat spike and spikelet development

#### Spike development

From the beginning of the wheat spike development, *LFY* expression is not uniform, with higher expression levels at the lower or leaf ridge than at the upper or spikelet ridge (Fig. 4A-C), a pattern reported previously in wheat by *in situ* hybridization (Shitsukawa et al., 2006). This spatial differentiation continues in the early spike development (W2.5) when *LFY* is abundant in the proximal region of the young SMs but rare in their distal region (Fig. 4B,C). A similar pattern was also reported in the SMs of young barley spikes (Zhong et al., 2021) and in the primary and secondary branch meristems in the developing rice panicles (Kyozuka et al., 1998; Miao et al., 2022). These results suggest a conserved *LFY* spatial pattern in developing grass inflorescences and highlight the importance of reduced *LFY* levels in initiating spikelet development.

In wheat, the SM regions with low *LFY* expression (Fig. 4B,C) show high levels of *VRN1* and *FUL2* transcripts (Fig. 5C,D) and, therefore, high *SQUAMOSA/LFY* ratios. We hypothesize that the change in the balance between SM-promoting and -repressing pioneer transcription factors is crucial for marking the regions where the lateral spikelets will develop. This hypothesis is also supported by the drastic changes in the relative smFISH signal densities of these genes in the IM and two youngest lateral meristems between the early stages of spike development (W2.5) and the time of the IM→TS transition (determined by the presence of *FZP*, W3.25; Fig. S4). In the IM, the *VRN1/LFY* ratio increased more than eightfold and the *FUL2/LFY* ratio increased more than 25-fold between W2.5 and W3.25 at the initiation of the terminal spikelet (Fig. S8, Table S8).

Changes in gene dosage for *LFY* (Fig. S1) or the *SQUAMOSA* genes result in opposite changes in SNS, confirming the importance of their relative transcript levels on spike development. The single *lfy-A* and *lfy-B* mutants show intermediate reductions in SNS relative to the combined *lfy* mutant (Fig. S1), whereas mutations in *FUL2* result in smaller increases in SNS than mutations in *VRN1*, which is expressed at higher levels than *FUL2* in the IM (Fig. S8A, Table S8) (Li et al., 2019). Interestingly, four recently cloned genes affecting SNS in wheat – *WAPO1* (Kuzay et al., 2022), *FT-A2* (Shaw et al., 2019; Glenn et al., 2022), *bZIPC1* (Glenn et al., 2023) and *SPL17* (Liu et al., 2023) – show potential connections with the regulation of *VRN1* or *LFY* (Fig. S12). In summary, we propose that the modulation of *VRN1* or *LFY* transcript levels in the IM plays an important role in the determination of SNS in wheat.

*LFY* was co-expressed with *WAPO1* in the IM at the early stages of spike development (Fig. 4A), but not at later stages (Fig. 4B,C, Fig. S5B,C). This early colocalization in the IM seems to be sufficient to explain the similar reduction in SNS observed in the *lfy* and *wapo1* mutants. Both mutants were associated with similar reductions in the rate of SM formation relative to the WT, rather than by a change in the timing of the IM→TS transition (Fig. 3C,D). Given that these differences were evident from the earliest stages of spikelet development, when both *LFY* and *WAPO1* were co-expressed in the IM (Fig. 4A), we hypothesize that the transient formation of the LFY-WAPO1 complex is sufficient to activate the gene expression networks that accelerate the rate of SM initiation.

#### Spikelet development

The smFISH studies also provided insights into the dynamic spatial distribution of *LFY*, *WAPO1*, and the floral organ identity genes during spikelet development. In the more-developed central spikelets at W3.25, *LFY* was highly expressed in a narrow nest-shaped region distal to the lemma primordia, delimiting a distal spikelet meristem region with lower *LFY* expression (Fig. 4D). This intense *LFY* expression band has been also observed distal to the lemma primordia of the second and third florets by *in situ* hybridization in more-developed wheat spikelets (Shitsukawa et al., 2006), highlighting its importance for normal floret development.

Within the distal region of the developing spikelets, *WAPO1* was co-expressed with *LFY* in multiple cells, including one or two cell layers within the region of high *LFY* expression (Fig. 4D, Fig. S6). Within this overlapping region LFY and WAPO1 proteins may have a higher chance to interact with each other, providing valuable spatial information to the floral organ identity genes. This hypothesis is indirectly supported by similar reductions in the expression levels of the floral organ identity genes and similar floral abnormalities in both the *wapo1* and *lfy* mutants (Fig. 1F, Fig. S11; Kuzay et al., 2022).

In *Arabidopsis*, the UFO-LFY complex regulates a different set of gene targets than LFY alone (Rieu et al., 2023b), and both genes are required for the correct regulation of the floral organ identity genes *AP3* (Chae et al., 2008; Rieu et al., 2023b) and *PI* (Honma and Goto, 2000). These results are consistent with the downregulation of the wheat floral organ identity genes in the *lfy* and *wapo1* mutants, and with the overlap between the expression domains of the wheat floral organ identity genes (Fig. 5, Fig. S10) and *LFY*-*WAPO1* co-expression region in the distal part of the wheat developing spikelets.

In summary, this study shows that *LFY* plays important roles in both floral organ and spike development. The dynamic spatial and temporal expression profiles of *LFY* and *WAPO1* in the developing spikelets correlate well with the function of these genes in the regulation of floral organ identity genes and normal floret development. In addition, these genes regulate the rate of SM formation, and interact with the *SQUAMOSA* genes in the regulation of SNS. Therefore, natural or induced variation in these genes can be used to improve this important agronomic trait in a crop that is central for global food security.

## MATERIALS AND METHODS

### Ethyl methanesulfonate-induced *LFY* mutants and their interactions with *VRN1*, *FUL2* and *WAPO1*

We screened the sequenced tetraploid wheat variety Kronos population (Krasileva et al., 2017) by BLASTN to identify loss-of-function mutations in the *LFY-A1* and *LFY-B1* homeologs. To reduce background mutations, the *lfy-B* mutant was backcrossed twice to Kronos and then to the *lfy-A* mutant. The double mutant was backcrossed once to Kronos and, among the progeny, homozygous sister lines were selected for the four possible homozygous combinations, including the *lfy-A lfy-B* combined mutant (here termed *lfy*). This line is BC_1_ for *lfy-A* and BC_2_ for *lfy-B* so it is referred to as BC_1-2_. Genome-specific markers for the *lfy-A* and *lfy-B* mutations were designed and used to genotype plants during backcrossing and combination with other mutations described below. Primers are listed in Table S1.

To study the interactions between *LFY* and other spike development genes, we intercrossed the *lfy* combined mutant with previously developed Kronos lines homozygous for loss-of-function ethyl methanesulfonate or CRISPR mutations in both genomes of *VRN1* (Chen and Dubcovsky, 2012), *FUL2* (Li et al., 2019) or *WAPO1* (Kuzay et al., 2022). These lines had at least two backcrosses to the parental line Kronos to reduce the number of background mutations. For these crosses, we used a line heterozygous for one of the *LFY* mutations to restore fertility, and molecular markers to select the four possible homozygous combinations in the F_2_ progeny of each cross.

### Plant growth and phenotypic characterization

Plants were stratified for 2 days at 4°C in the dark and then planted in growth chambers (PGR15, Conviron, Manitoba, Canada). Lights were set to 350 μmol m^−2^ s^−1^ at canopy level. Plants were grown under inductive 16-h long days or non-inductive 8-h short days with temperatures set during the day to 22°C and during the night to 17°C. Relative humidity in growth chambers was maintained at 60-70% throughout the duration of the experiments. Heading time was recorded as the number of days from germination to full emergence of the spike from the leaf sheath. SNS was measured at maturity from the main tiller.

### Generation of the wheat transgenic lines overexpressing *LFY*

We cloned the *LFY-A* coding regions by PCR from cDNA derived from Kronos developing spikes using primer LFY-A-GW-F combined with LFY-A-GW-R2 (listed in Table S1). We then recombined it into the pDONR/zeo entry vector using Life Technologies BP Clonase II following the manufacturer's protocol. The pDONR/zeo vector containing the *LFY-A* coding region was next recombined into the Japan Tobacco pLC41 vector downstream of the maize *UBIQUITIN* promoter using Life Technologies LR Clonase II to generate a construct expressing *LFY* with a C-terminal 3xHA tag, which was verified by Sanger sequencing at each cloning step. The T-DNA binary construct was transformed into the wheat variety Kronos using *Agrobacterium-*mediated transformation (EHA105) at the UC Davis Plant Transformation Facility as described previously (Debernardi et al., 2020b). The presence of the *LFY* transgene was determined with primers LFY-Genotyping-R5 and UBI-F2 (listed in Table S1).

*LFY* transcript levels were determined by qRT-PCR using primers LFY_qPCR_F and LFY_qPCR_R and *ACTIN* as endogenous control (Table S1). For qRT-PCR experiments, RNA was extracted using the Spectrum Plant Total RNA Kit (Sigma-Aldrich) or as previously described by Ream et al. (2014). One μg of RNA was treated with RQ1 RNase-Free DNase (Promega, M6101) first and then used for cDNA synthesis with the High-Capacity cDNA Reverse Transcription Kit (Applied Biosystems). The qRT-PCR experiments were performed using Quantinova SYBR Green PCR kit (QIAGEN, 208052) in a 7500 Fast Real-Time PCR system (Applied Biosystems). Transcript levels for all genes are expressed as linearized fold-*ACTIN* levels calculated by the formula 2^(*ACTIN* CT−*TARGET* CT)^±s.e.m.

### smFISH

We used the Molecular Cartography™ technology from Resolve BioSciences, which is based on combinatorial smFISH. Wheat shoot apical meristems were collected from the vegetative to the spike lemma primordia stage. The samples were immediately fixed in 4% paraformaldehyde after harvest, dehydrated, and embedded in paraffin. Sections from the central plane of the developing spikes (10 µm thick) were placed on the slides and dried overnight at 37°C, followed by a 10-min bake at 50°C. The sections were then deparaffinized, permeabilized, and refixed according to the Resolve BioSciences user guide. After complete dehydration, the sections were mounted using SlowFade-Gold Antifade reagent, covered with a thin glass coverslip, and sent to Resolve BioSciences on dry ice for analysis as described in our previous study (Glenn et al., 2023).

Probes were designed using Resolve BioSciences' proprietary design algorithm and gene annotations from Chinese Spring RefSeqv1.1. To identify potential off-target sites, searches were confined to the coding regions. Each target sequence underwent a single scan for all k-mers, favoring regions with rare k-mers as seeds for full probe design. For each of the wheat genes selected for smFISH probe design (Table S9), we selected the homoeolog expressed at higher levels in a Kronos transcriptome including different stages of spike development (VanGessel et al., 2022), and provided Resolve BioSciences with their respective homeologs to be excluded in their quality control for primer specificity performed against all the coding sequences of the wheat genome (Ref Seq v1.1). Therefore, probes are not genome specific and may detect both homeologs for each gene (catalog number PGGS; all these probes are part of kit number K7128).

Imaging and image processing was performed as described previously (Glenn et al., 2023). Final image analysis and quantification of dot density was performed in ImageJ using the Polylux tool plugin to examine specific Molecular Cartography™ signals.

### Co-IP assay and western blotting

To examine the physical interaction between WAPO and LFY, we performed Co-IP experiments in wheat leaf protoplasts using a method described previously (Zhang et al., 2023), with minor modifications. The *WAPO1* coding region was initially synthesized by GENEWIZ into the pUC57 vector, amplified with primers WAPO1_BP_F and WAPO1_BP_R (Table S1), cloned into pDONR/zeo vector using Life Technologies BP Clonase II, and recombined into the Japan Tobacco pLC41 vector downstream of the maize *UBIQUITIN* promoter with a C-terminal 4xMYC tag (for transgenic experiments). Next, we switched both *UBI:WAPO1-MYC* and *UBI:LFY-HA* from the pLC41 binary vector to the smaller pUC19 vector to enhance the transfection efficiency of the protoplasts. The *UBI:WAPO-MYC* and *UBI-LFY-HA* DNA fragments were cleaved using restriction enzymes *Hind*III and *Spe*I, gel purified, and then ligated with the *Hind*III-*Xba*I linearized pUC19 vector (*Spe*I and *Xba*I create compatible ends). Both constructs were verified by digestions with restriction enzymes and Sanger sequencing.

We transformed Kronos leaf protoplasts with 50 μg of each of the *UBI:LFY-HA* and *UBI: WAPO1-MYC* plasmids in 50 ml tubes containing 2 ml of protoplast (roughly 0.5×10^6^ cell per ml). As negative controls, we performed separate transformations including only one of the two plasmids. After transformation, protoplasts were resuspended in 5 ml W5 buffer and incubated in a 6-well plate at room temperature overnight. Total protein was extracted with 1 ml of IP lysis buffer [25 mM Tris-HCl pH7.5, 150 mM NaCl, 1 mM EDTA, 1% NP-40 substitute, 5% glycerol and 1×protease inhibitors (Millipore Sigma, P9599)]. Part of the protein extract was set aside as input control (50 μl), and the rest was used for Co-IP using Pierce Anti-HA Magnetic Beads (Thermo Fisher Scientific, 88836) by gentle agitation on a tube rotator for 30 min at room temperature with additional 1× proteinase inhibitors (Millipore Sigma, P9599). Proteins were washed twice with 300 μl 1× TBS (25 mM Tris, 0.15 M NaCl at pH 7.5) with 0.05% Tween-20, and once with ultra-pure water before they were eluted by boiling the beads in 50 μl 1× Laemmli sample buffer for 10 min.

For western blotting, half of the Co-IP elution and 50 μg of input for each sample were loaded onto a 12% SDS-PAGE gel. After proteins were transferred to a PVDF membrane using the Bio-Rad Trans-Blot Turbo Transfer System (1704150), the membrane was blocked with 1× TBST buffer (20 mM Tris, 0.15 M NaCl, 0.1% Tween-20 at pH 7.5) containing 5% non-fat milk for 1 h at room temperature. Anti-cMyc-peroxidase monoclonal antibody (Roche, 11814150001) was added at a dilution of 1:10,000 and was incubated at room temperature for 1 h. After four 10 min washes using 1× TBST buffer, the signals were developed using SuperSignal West Femto Maximum Sensitivity Substrate (Thermo Fisher Scientific, 34096). After imaging, the membrane was stripped with mild stripping buffer (1.5% glycine, 0.1% SDS, 1% Tween 20, pH 2.2), re-blocked for 1 h at room temperature, and then probed with anti-HA-peroxidase at a dilution of 1:2500 (Roche, 12013819001) for 1 h at room temperature.

## Supplementary Material



10.1242/develop.202803_sup1 Supplementary information

Table S1. Primers used in this study.

Table S2.

Table S3. Supporting data for Fig. S1. Effect of individual and combined LFY mutation on SNS.

Table S4. Supporting Data for Fig. S2. Transcript levels of UBI:LFY-HA in leaves reltive to non-transgenic sister lines and Kronos. The LFY-A homeolog was used in these transgenic lines.

Table S5. Supporting data for Fig. 2. Effect of UBI:LFY-HA on spikelet number per spike (SNS) in Kronos wildtype (WT) and lfy mutant.

Table S6. Supporting data for Fig. 3. Genetic interactions for spikelet number per spike (SNS) between lfy and wapo1 loss-of-function mutants, and rate of spikelet meristem development (Fig. 3C-D).

Table S7. Supporting data for Fig. S3. LFY expression in wheat from previously published RNAseq studies.

Table S8. Changes in the hybridization signal density of LFY, VRN1, FUL2 and FZP at the distal region of the developing spike between the double ridge (W2.5) and the lemma primordia stage (W3.25). Data supporting Fig. S8.

Table S9. Genes used in the sm-FISH experiments. Rice homologs and wheat gene identification numbers in Chinese Spring RefSeq v1.1.

Table S10. Supporting data for Fig. S7. qRT-PCR expression of VRN1 and FUL2 in wildtype (WT) Kronos and loss-of-function lfy mutant.

Table S11. Supporting data for Fig. S11. qRT-PCR expression of MADS-box genes involved in floral development in wildtype (WT) Kronos and loss-of-function lfy mutant.

Table S12. Supporting data for Fig. 6. Genetic interactions for SNS between LFY and VRN1 and between LFY and FUL2.
