# Supplementary material for: LEAFY and WAPO1 jointly regulate spikelet number per spike and floret development in wheat
Source: Development. 2024 Jul 31;151(15):dev202803. doi: 10.1242/dev.202803 (PMC11317094; doi:10.1242/dev.202803)
Supplement: Supplementary information [file develop-151-202803-s1.pdf]

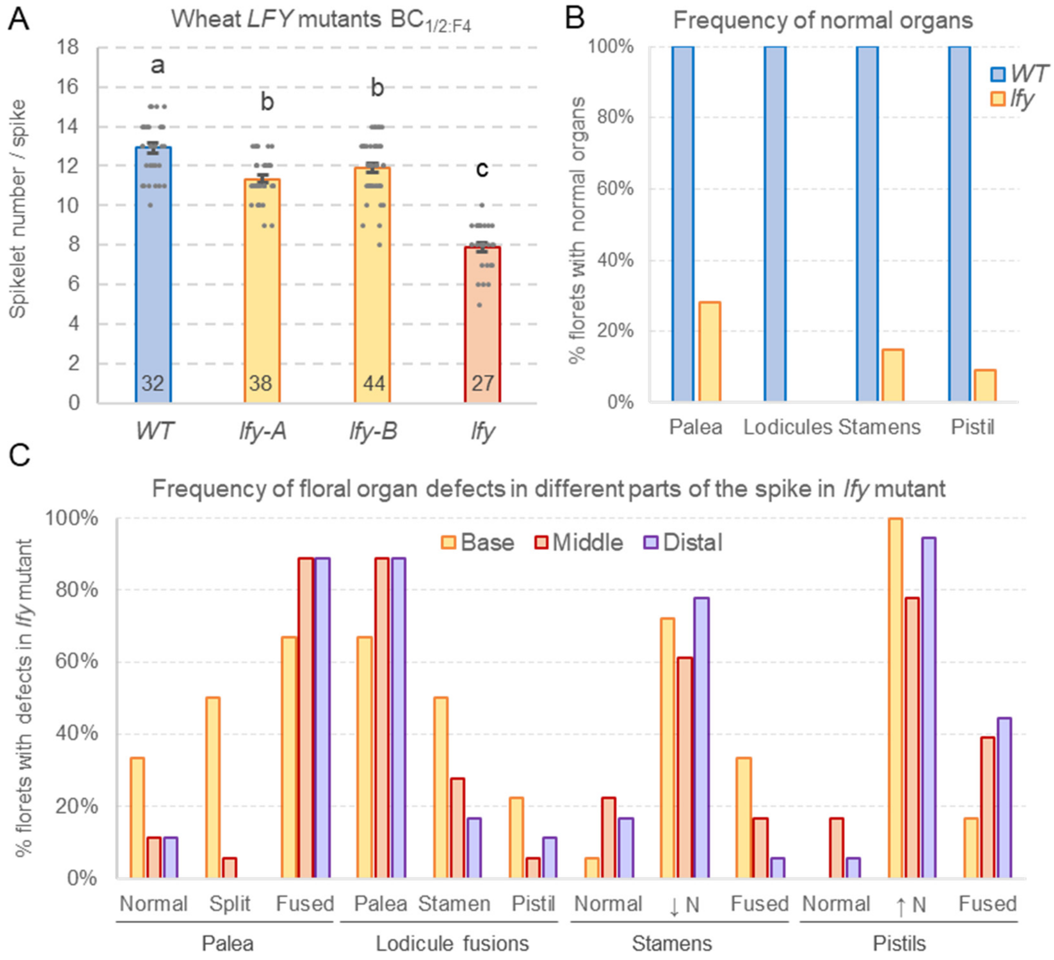

Fig. S1. Effect of *LFY* mutants on spikelet number per spike (SNS) and floral defects. (**A**) Effect of single (*lfy-A* and *lfy-B*) and combined *lfy* mutants on SNS. Numbers inside the bars indicate number of individual plants measured. Different letters on top of the bars indicate significant differences based on two-tail Tukey test ($P < 0.05$). Raw data and statistics are available in Table S3. (**B**) Frequency of florets with normal and abnormal floral organs in Kronos wildtype and *lfy* mutant (n = 54 florets). Abnormalities include increased or reduced number, morphological changes and fusions with other organs. (**C**) Frequency of abnormalities in the different floral organs in *lfy* mutant, based on 18 florets from the base of the spike, 18 from the middle and 18 from the distal part of the spike. Half of the florets correspond to the 1st floret and the other half to the 2nd floret in the selected spikelets. Raw data are available in Table S2.

A

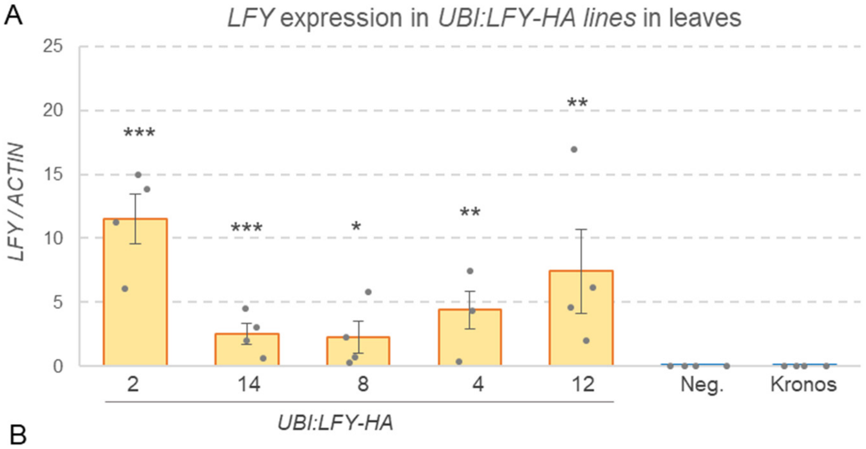

B

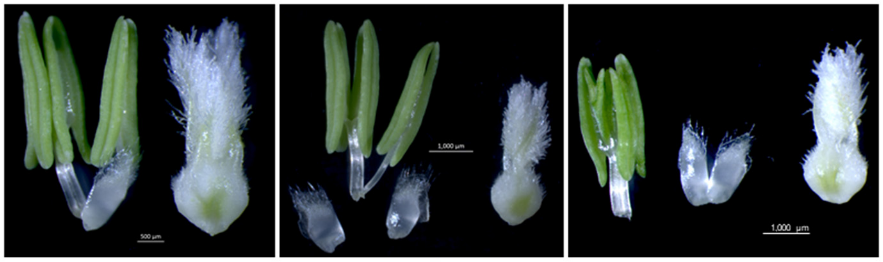

C

Floret	Lodicule	Stamen	Pistil
1	1 & fused	Fused filaments	Extra stigmas
2	N	N	N
3	N	N	N
4	N	Fused filaments	Extra stigmas
5	N	N	N
6	N	N	N
7	N	N	N
8	N	N	N
9	N	Fused filaments	N
10	N	Fused filaments	Extra stigmas
11	Absent	Fused filaments	Extra stigmas
12	Absent	Fused filaments	Extra stigmas
13	N	N	N
14	N	N	N
% normal	78.6%	57.1%	64.3%

Fig. S2. Transgenic plants expressing *LFY* under the *UBIQUITIN* promoter.
(**A**) *LFY* transcript levels in the leaves of transgenic plants overexpressing *LFY*. The *LFY-A* coding region fused with an HA tag was driven by the maize *UBIQUITIN* promoter (*UBI:LFY-HA*). Five independent events were evaluated. Note the absence of expression of *LFY* in the negative transgenic sister lines (Neg.) and the wildtype Kronos control. *P* values are two-tail *t*-tests of each transgenic event (n = 4) relative to the combined Neg. and wildtype Kronos control (n = 8). * $P = 0.05$, ** $P = 0.01$, *** $P = 0.001$. Error bars are SEM. Raw data and statistics are available in Table S4. (**B**) Floral defects in *UBI:LFY-HA* included reduced number or fused lodicules, fused anther filaments and extra stigmas. (**C**) Frequency of floral defects in 14 florets. Note the higher proportion of normal plants compared to the *lfy* mutants in Fig. S1B.

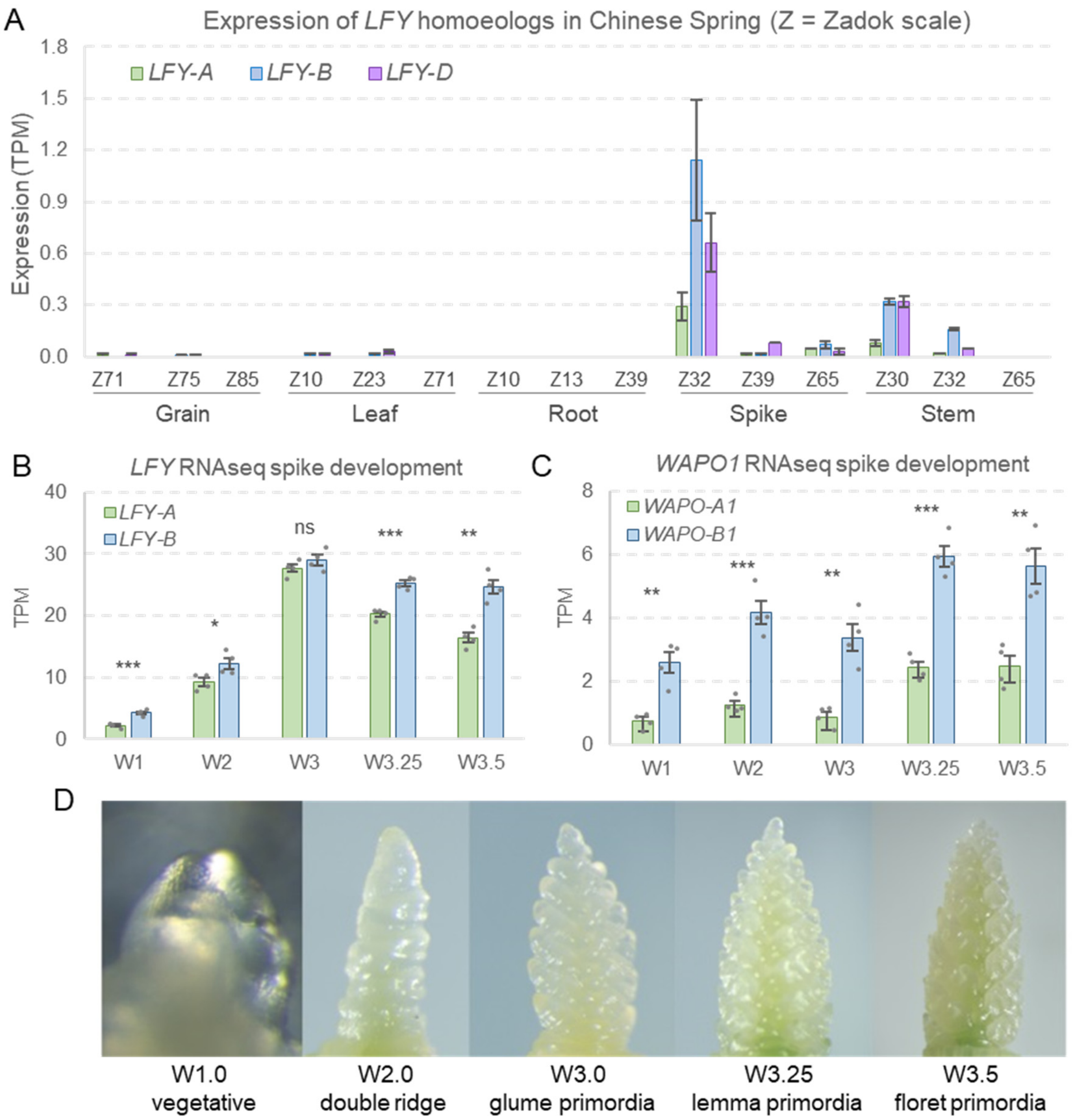

Fig. S3. Expression analysis of *LFY* and *WAPO1*. (**A**) *LFY* RNA-seq data for Chinese Spring across five tissues and three developmental stages (Choulet *et al.*, 2014). (**B**) *LFY* and (**C**) *WAPO1* transcript levels during early stages of Kronos spike development (VanGessel *et al.*, 2022). (**D**) Examples of developing spikes at the different spike development stages collected in the Kronos RNA-seq study (VanGessel *et al.*, 2022). W numbers indicate values in the Waddington scale of wheat spike development (Waddington *et al.*, 1983). TPM= transcripts per million. Averages are based on four biological replications per stage in each experiment and error bars are SEM. *P* values are from *t*-tests between homoeologs. ns= not significant, * = $P < 0.05$ and ** = $P < 0.01$. Raw data and statistics are available in Table S7.

Development • Supplementary information

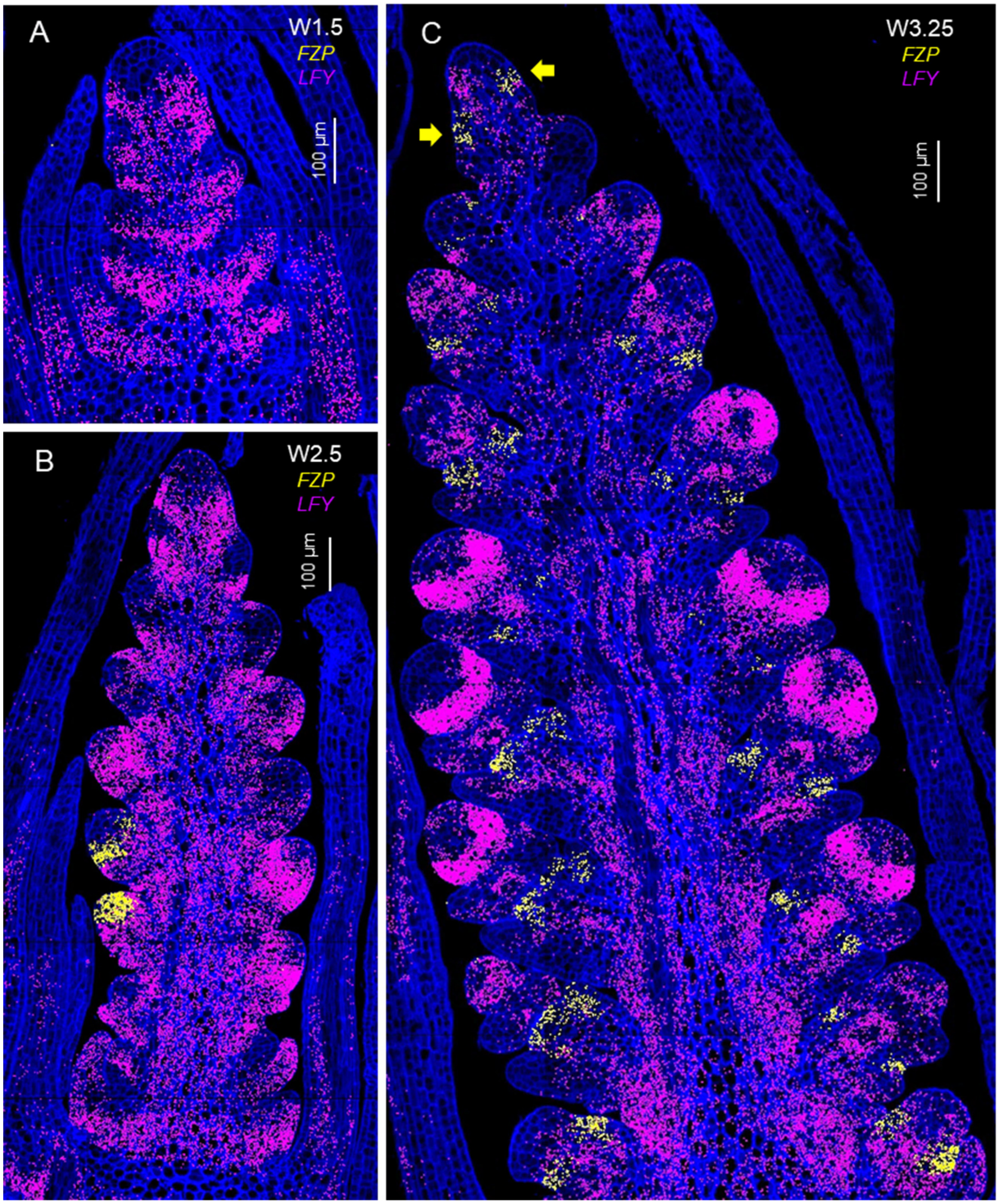

Fig. S4. Single-molecule fluorescence *in situ* hybridization (smFISH) for *FRIZZY PANICLE* (*FZP*). *FZP* (*TraesCS2A02G116900*) is used as a marker for the initiation of spikelet development. *FZP* transcripts are indicated by yellow dots and *LFY* by purple dots. Cell walls stained with calcofluor are in dark blue. (**A**) Elongated shoot apical meristem transitioning to the reproductive stage (W1.5). (**B**) Late double ridge (DR, W2.5). (**C**) Lemma primordia (LP, W3.25). Yellow arrows indicate *FZP* expression in the youngest lateral meristems, suggesting a transition to glumes and the start of the IM transition to a terminal spikelet. Quantification of the *FZP* signal is available in Table S8.

Development • Supplementary information

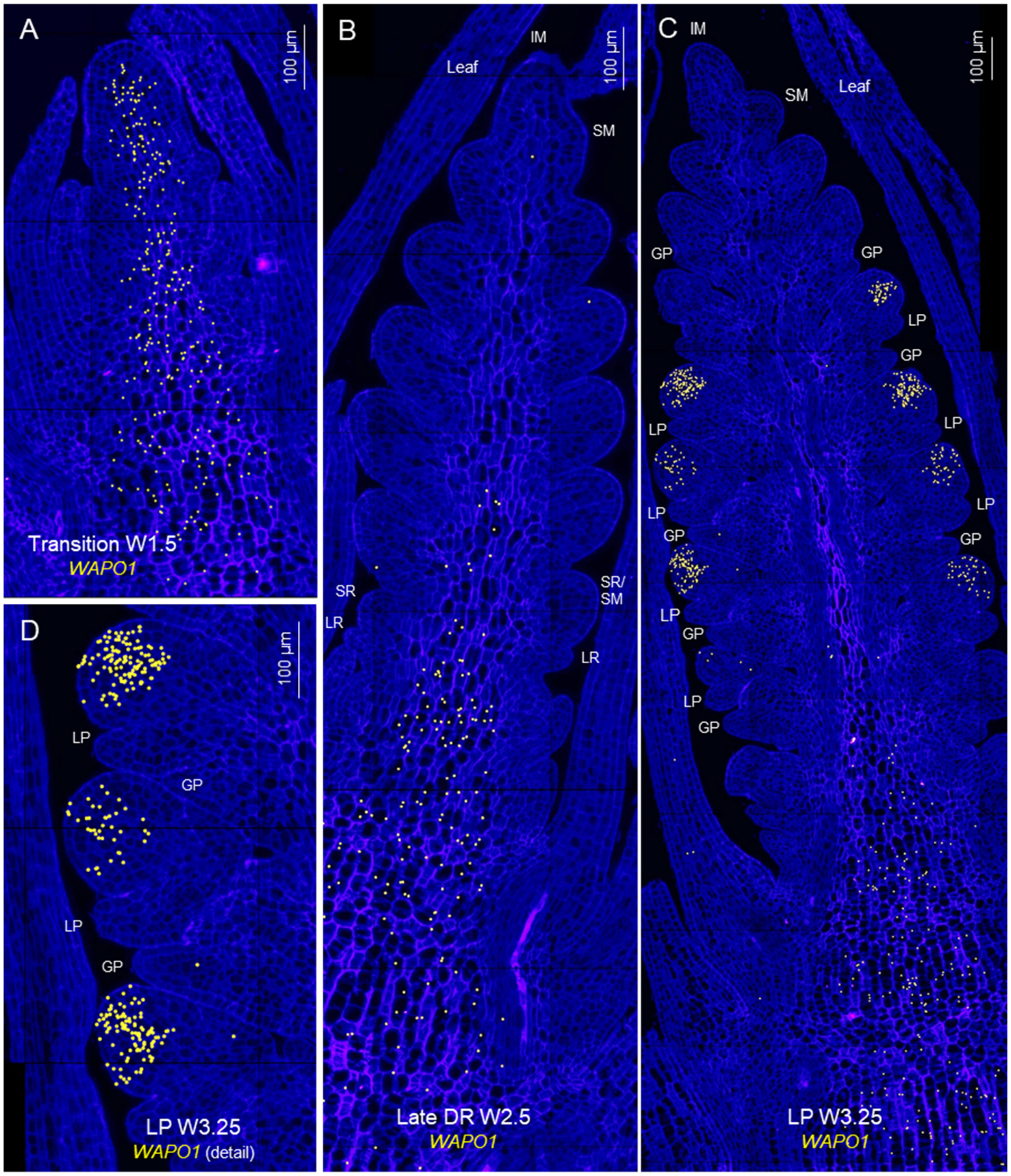

Fig. S5. Single-molecule fluorescence *in situ* hybridization (smFISH) for *WAPO1*. *WAPO1* (*TraesCS7B02G384000*) transcripts are indicated with yellow dots. Cell walls stained with calcofluor are presented in dark blue. (**A**) Elongated shoot apical meristem transitioning to the reproductive stage (W1.5). (**B**) Late double ridge (DR) stage (W2.5). (**C**) Lemma primordia (LP) stage (W3.25). (**D**) Detail of developing spikelet in C. LR = leaf ridge, SR = spikelet ridge, GP= glume primordium, LP= lemma primordium, IM = inflorescence meristem, SM= spikelet meristem (only one labelled). Scale bars are 100 µm.

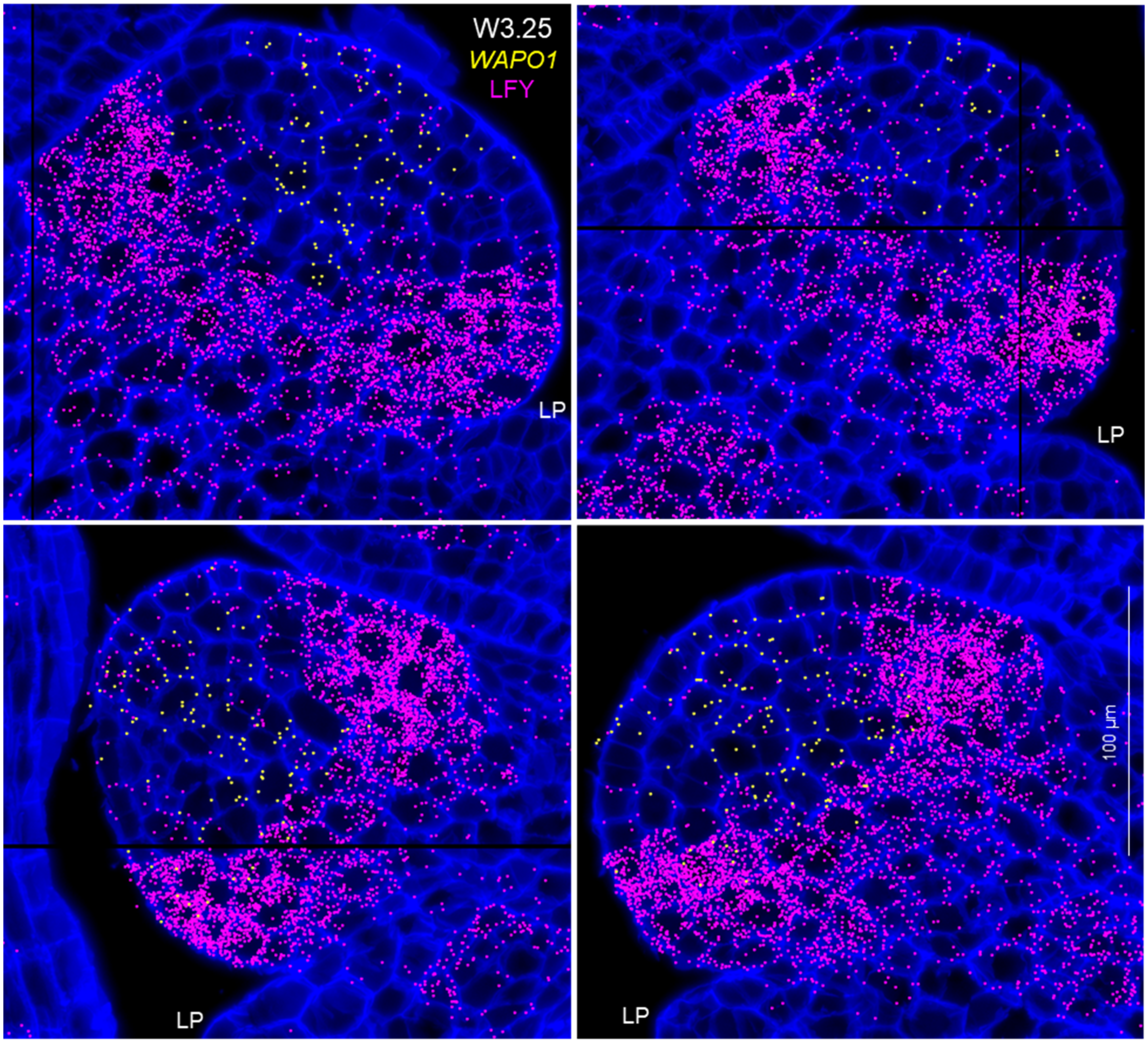

Fig. S6. Single-molecule fluorescence *in situ* hybridization (smFISH) showing detail of the overlap between *LFY* and *WAPO1* in spikelet meristems. Spikelet meristems at the lemma primordia stage at W3.25. *WAPO1* transcripts are indicated with yellow dots and *LFY* transcript with purple dos. Cell walls stained with calcofluor are presented in dark blue. LP= lemma primordium. Scale bars are 100 µm.

Development • Supplementary information

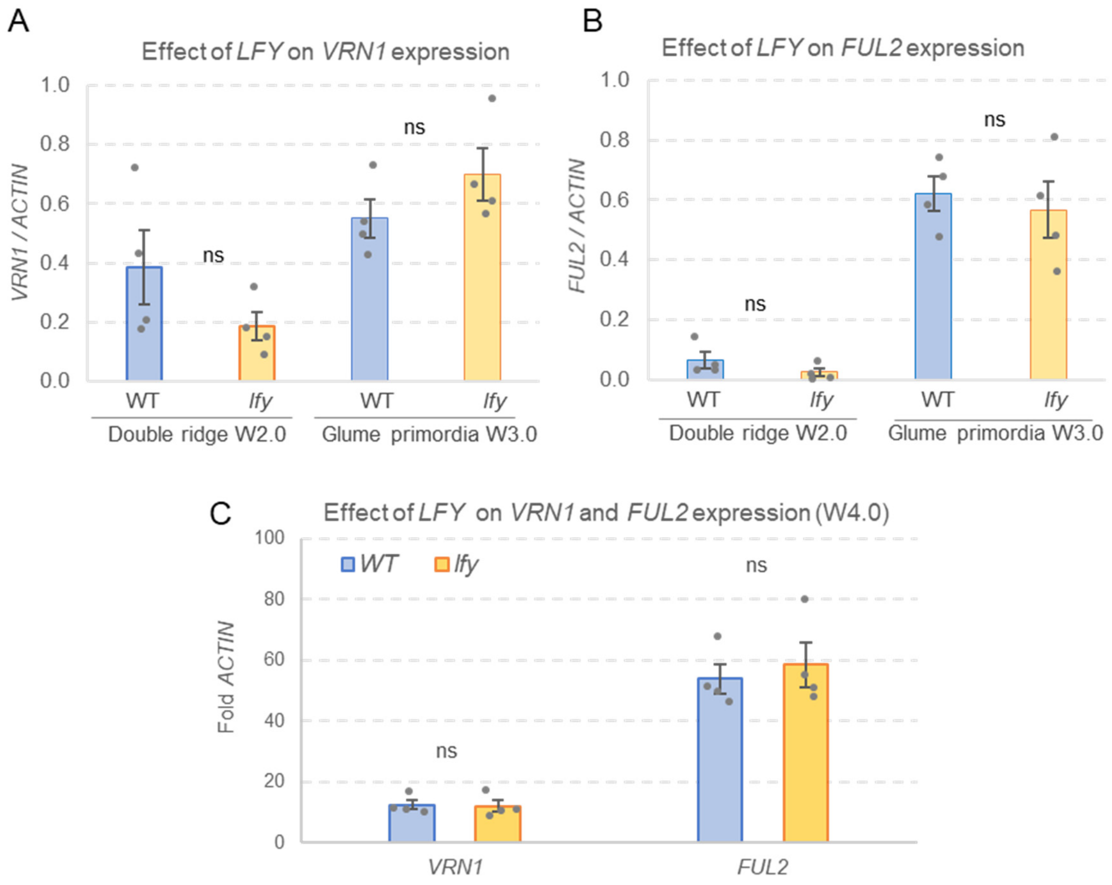

Fig. S7. Effect of *lfy* mutations on *VRN1* and *FUL2* expression. Transcript levels were estimated by qRT-PCR relative to *ACTIN* endogenous control. (**A**) *VRN1* transcript levels at W.2.0 and W3.0 (**B**) *FUL2* transcript levels at W.2.0 and W3.0. (**C**) *VRN1* and *FUL2* transcript levels at W4.0. Bars are averages of four biological replicates and error bars are SEM. Each biological replicate represents one RNA extraction from a pool of four apices from Kronos wildtype (WT) and *lfy* mutant at the double ridge (W2.0), glume primordia (W3.0) or stamen primordium (4.0) stages of spike development. ns = not significant. Raw data and statistics are available in Table S10.

Development • Supplementary information

A

TPM A+B	W1.0	W2.0	W3.0	W3.25	W3.5
VRN1	9.27	33.70	52.48	51.16	46.81
FUL2	0.44	8.90	33.89	42.83	45.20
FUL3	0.83	10.37	18.93	20.02	19.12
LFY	6.42	21.54	56.68	45.51	41.04

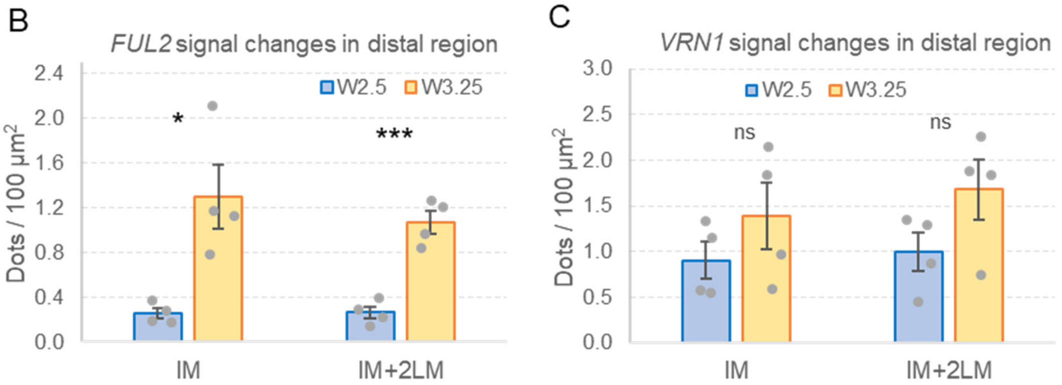

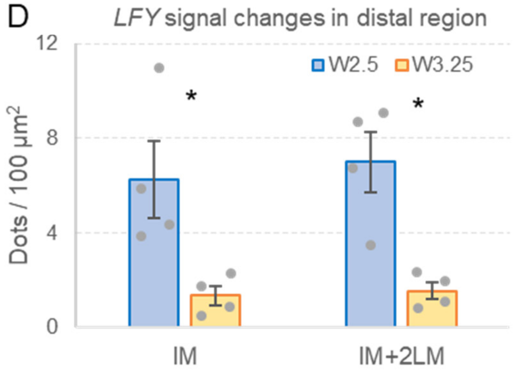

E smFISH signal ratio changes in distal region

Ratio	*FUL2 / LFY*		*VRN1 / LFY*	
Spike stage	IM	IM+2LM	IM	IM+2LM
W2.5	4%	4%	15%	14%
W3.25	114%	80%	123%	115%

Fig. S8. Changes in *VRN1*, *FUL2* and *LFY* between W2.5 and W3.25. (**A**) *VRN1*, *FUL2*, *FUL3* and *LFY* transcripts per million (TPM) from developing spikes at five stages (VanGessel *et al.*, 2022). Values are the sum of A and B genome averages of four replications. Values for each genome are available in Table S8. (**B-D**) Changes in hybridization signal density between late double ridge stage (W2.5) and lemma primordia stage (W3.25) at the inflorescence meristem (IM) and the IM plus the two youngest lateral meristems (IM+2LM). (**B**) *FUL2*, (**C**) *VRN1*, (**D**) *LFY*. (**E**) *FUL2/LFY* and *VRN1/LFY* ratios between hybridization signals at the IM and IM+2LM at W2.5 and W3.25. Values are averages of four sections at each stage (smFISH figures in Table S8) and error bars are s.e.m. ns = not significant, * = $P < 0.05$ and *** = $P < 0.001$. Raw data and statistics are available in Table S8.

Development • Supplementary information

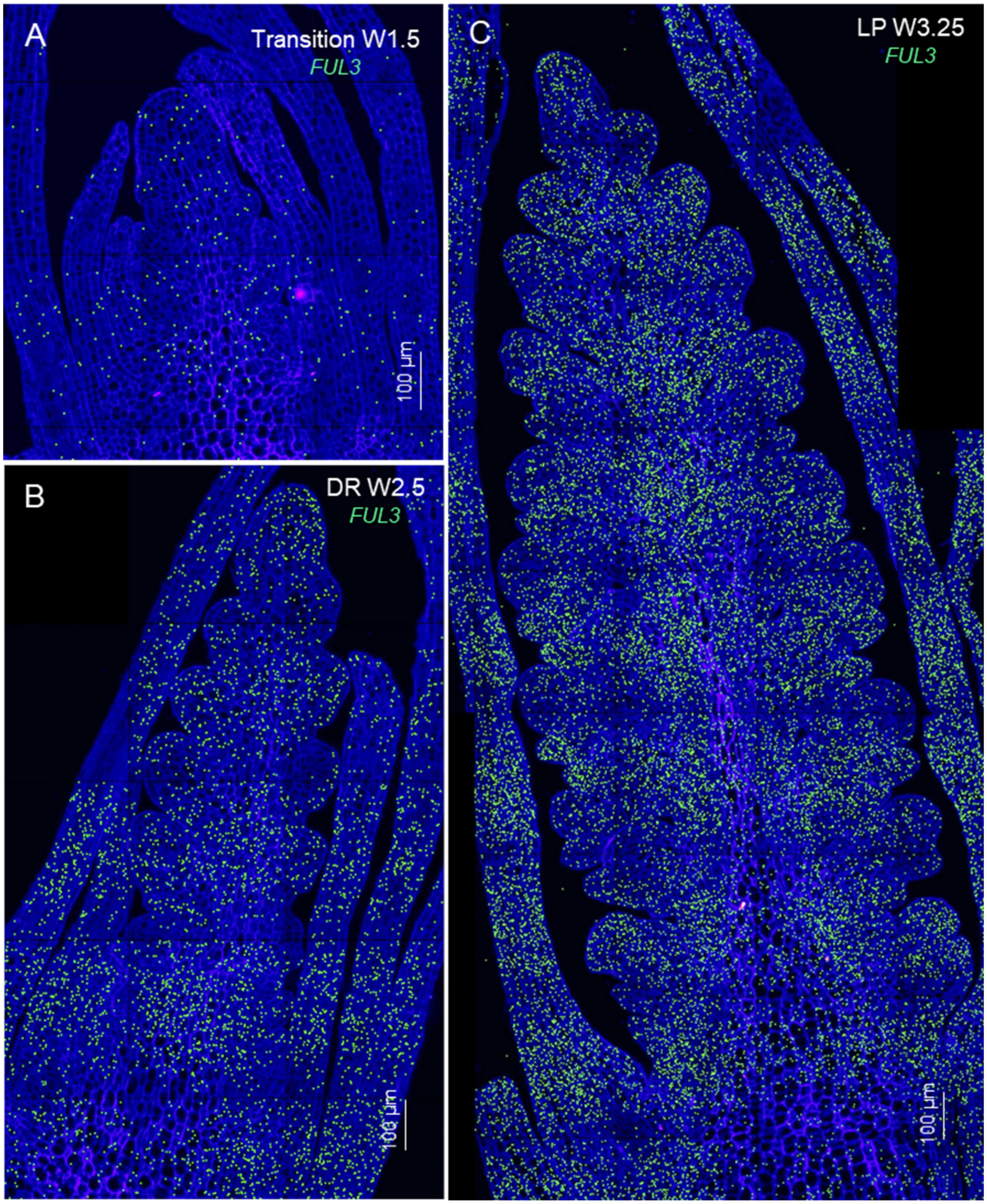

Fig. S9. Single-molecule fluorescence *in situ* hybridization (smFISH) for *FUL3*. Cell walls stained with calcofluor are presented in dark blue. (**A**) Elongated shoot apical meristem transitioning to the reproductive stage (W1.5). (**B**) Late double ridge stage (W2.5). (**C**) Lemma primordia stage (W3.25). Scale bars are 100 µm.

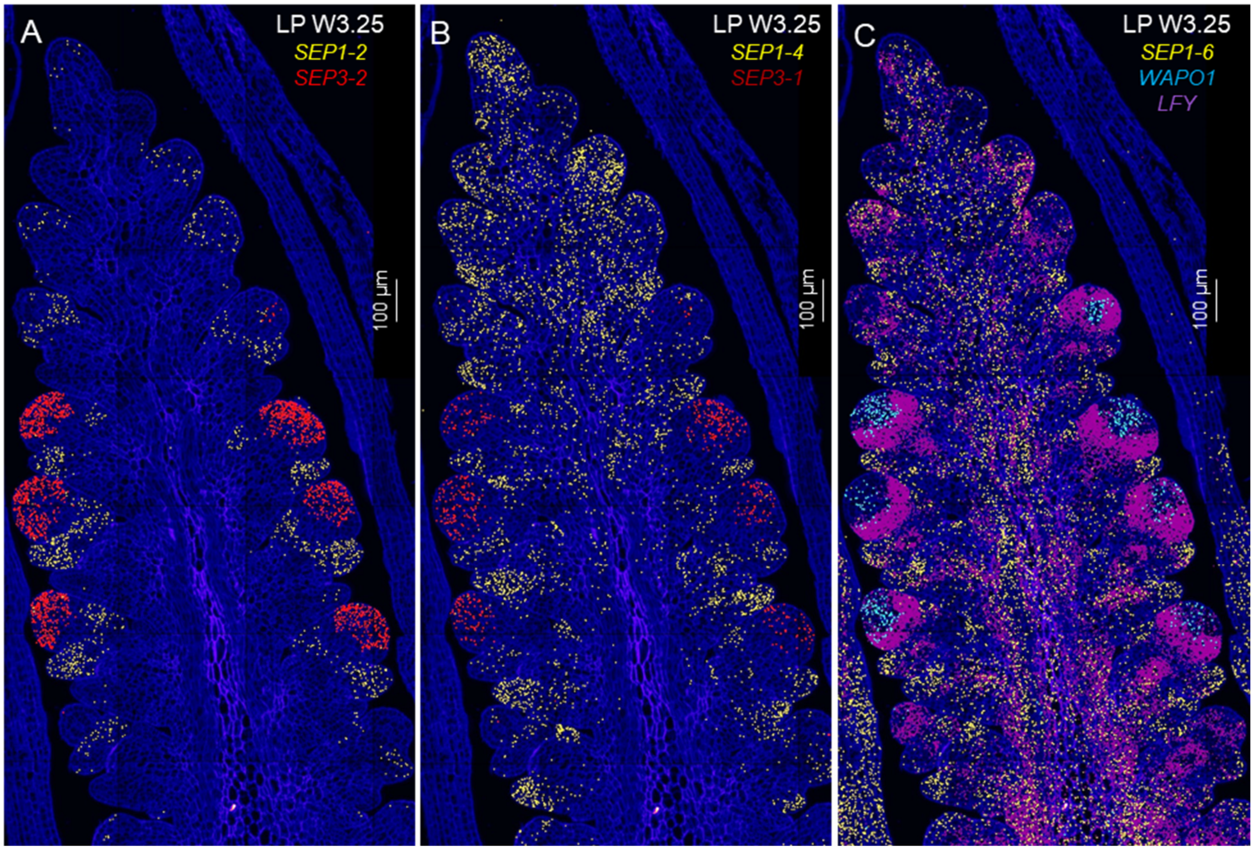

Fig. S10. Single-molecule fluorescence *in situ* hybridization (smFISH) for *SEPALLATA* genes. Only the lemma primordia stage (W3.25) is presented. Cell walls stained with calcofluor are presented in dark blue. (**A**) *SEP1-2* (yellow) and *SEP3-2* (red). (**B**) *SEP1-4* (yellow) and *SEP3-1* (red). (**C**) *SEP1-6* (yellow), *WAPO1* (light blue) and *LFY* (violet). Scale bars are 100 µm. Gene identifications based on CS RefSeq v1.1 and rice orthologs are provided in Table S9.

Development • Supplementary information

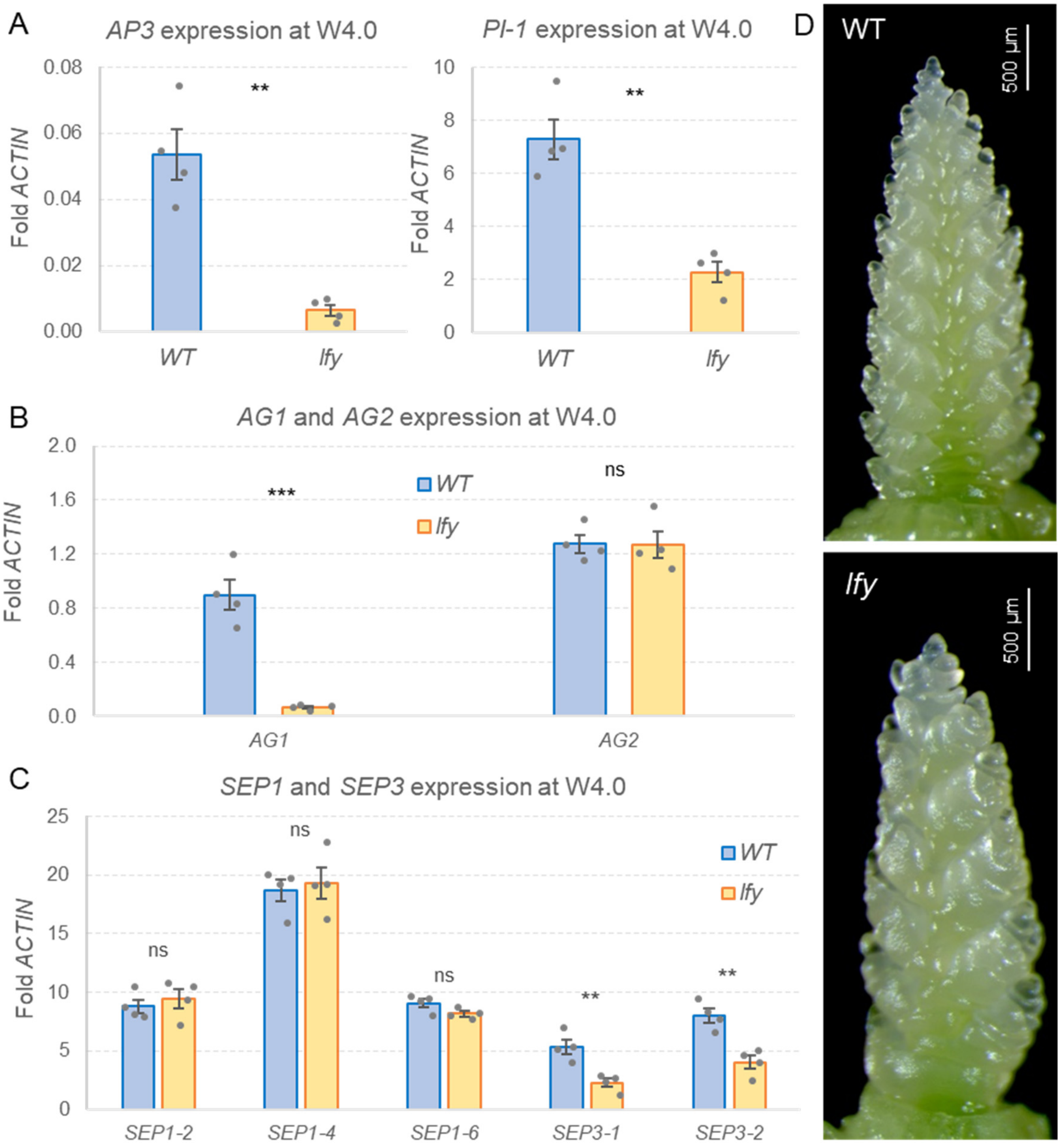

Fig. S11. Effect of *lfy* mutations on the expression of floral organ identity genes. Expression of class-B, -C, and -E MADS-box genes was characterized in wheat developing spikes at the stamen primordia stage (W4.0) using qRT-PCR with *ACTIN* as endogenous control. (**A**) class-B genes *AP3-1* and *PI1*, (**B**) class-C genes *AG1* and *AG2*, (**C**) class-E genes *SEP1-2*, *SEP1-4*, *SEP1-6*, *SEP3-1*, *SEP3-2*. (**D**) Representative wheat developing spikes at W4.0 in WT Kronos and *lfy* mutants (note the reduced SNS). Bars are averages of four biological replicates and error bars are standard errors of the means (SEM). Each biological replicate represents one RNA extraction from a pool of four apices from Kronos wildtype (WT) and *lfy* mutant. ns = not significant, ** $P < 0.01$, *** $P < 0.001$. Raw data and statistics are available in Table S11.

Development • Supplementary information

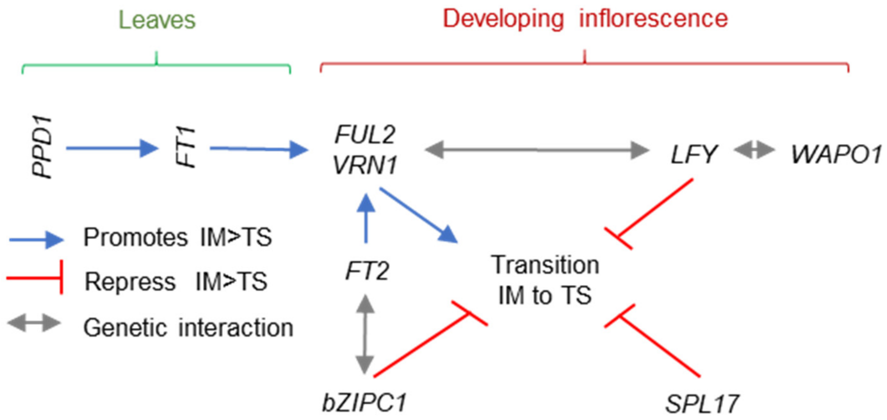

Fig. S12. Simplified working model of the regulation of SNS in wheat. The negative effect of *bZIPC1* in the regulation of the transition between the inflorescence meristem to a terminal spikelet (IM>TS), and its interaction with *FT2* is based on (Glenn *et al.*, 2023), the effect of *SPL17* on SNS in wheat is based on (Liu *et al.*, 2023). The positive effects of *FT1* and *FT2* on the regulation of *VRN1* are based on (Li and Dubcovsky, 2008, Lv *et al.*, 2014, Li *et al.*, 2019, Shaw *et al.*, 2019), and the positive effect of *PPD1* in the regulation of *FT1* and *FT2* on (Shaw *et al.*, 2013).

Development • Supplementary information

Table S1. Primers used in this study.

Available for download at
https://journals.biologists.com/dev/article-lookup/doi/10.1242/dev.202803#supplementary-data

Table S2.

Available for download at

https://journals.biologists.com/dev/article-lookup/doi/10.1242/dev.202803#supplementary-data

Table S3. Supporting data for Fig. S1. Effect of individual and combined LFY mutation on SNS.

Available for download at
https://journals.biologists.com/dev/article-lookup/doi/10.1242/dev.202803#supplementary-data

Table S4. Supporting Data for Fig. S2. Transcript levels of UBI:LFY-HA in leaves reltive to non-transgenic sister lines and Kronos. The LFY-A homeolog was used in these transgenic lines.

Available for download at
https://journals.biologists.com/dev/article-lookup/doi/10.1242/dev.202803#supplementary-data

Table S5. Supporting data for Fig. 2. Effect of UBI:LFY-HA on spikelet number per spike (SNS) in Kronos wildtype (WT) and lfy mutant.

Available for download at
https://journals.biologists.com/dev/article-lookup/doi/10.1242/dev.202803#supplementary-data

Table S6. Supporting data for Fig. 3. Genetic interactions for spikelet number per spike (SNS) between lfy and wapo1 loss-of-function mutants, and rate of spikelet meristem development (Fig. 3C-D).

Available for download at
https://journals.biologists.com/dev/article-lookup/doi/10.1242/dev.202803#supplementary-data

Table S7. Supporting data for Fig. S3. LFY expression in wheat from previously published RNAseq studies.

Available for download at
https://journals.biologists.com/dev/article-lookup/doi/10.1242/dev.202803#supplementary-data

Table S8. Changes in the hybridization signal density of LFY, VRN1, FUL2 and FZP at the distal region of the developing spike between the double ridge (W2.5) and the lemma primordia stage (W3.25). Data supporting Fig. S8.

Available for download at
https://journals.biologists.com/dev/article-lookup/doi/10.1242/dev.202803#supplementary-data

Table S9. Genes used in the sm-FISH experiments. Rice homologs and wheat gene identification numbers in Chinese Spring RefSeq v1.1.

Available for download at
https://journals.biologists.com/dev/article-lookup/doi/10.1242/dev.202803#supplementary-data

Table S10. Supporting data for Fig. S7. qRT-PCR expression of VRN1 and FUL2 in wildtype (WT) Kronos and loss-of-function lfy mutant.

Available for download at
https://journals.biologists.com/dev/article-lookup/doi/10.1242/dev.202803#supplementary-data

Table S11. Supporting data for Fig. S11. qRT-PCR expression of MADS-box genes involved in floral development in wildtype (WT) Kronos and loss-of-function lfy mutant.

Available for download at
https://journals.biologists.com/dev/article-lookup/doi/10.1242/dev.202803#supplementary-data

Table S12. Supporting data for Fig. 6. Genetic interactions for SNS between LFY and VRN1 and between LFY and FUL2.

Available for download at
https://journals.biologists.com/dev/article-lookup/doi/10.1242/dev.202803#supplementary-data

References for Supplemental Figures

Choulet, F., Alberti, A., Theil, S., Glover, N., Barbe, V., Daron, J., Pingault, L., Sourdille, P., Couloux, A., Paux, E., Leroy, P., Mangenot, S., Guilhot, N., Le Gouis, J., Balfourier, F., Alaux, M., Jamilloux, V., Poulain, J., Durand, C., Bellec, A., Gaspin, C., Safar, J., Dolezel, J., Rogers, J., Vandepoele, K., Aury, J.M., Mayer, K., Berges, H., Quesneville, H., Wincker, P. and Feuillet, C. (2014) Structural and functional partitioning of bread wheat chromosome 3B. *Science*, **345**, 1249721.

Glenn, P., Woods, D.P., Zhang, J., Gabay, G., Odle, N. and Dubcovsky, J. (2023) Wheat bZIPC1 interacts with FT2 and contributes to the regulation of spikelet number per spike. *Theor. Appl. Genet.*, **136**, 237.

Li, C. and Dubcovsky, J. (2008) Wheat FT protein regulates *VRN1* transcription through interactions with FDL2. *Plant J.*, **55**, 543-554.

Li, C., Lin, H., Chen, A., Lau, M., Jernstedt, J. and Dubcovsky, J. (2019) Wheat *VRN1*, *FUL2* and *FUL3* play critical and redundant roles in spikelet development and spike determinacy. *Development*, **146**, dev175398.

Liu, Y.Y., Chen, J., Yin, C.B., Wang, Z.Y., Wu, H., Shen, K.C., Zhang, Z.L., Kang, L.P., Xu, S., Bi, A.Y., Zhao, X.B., Xu, D.X., He, Z.H., Zhang, X.Y., Hao, C.Y., Wu, J.H., Gong, Y., Yu, X.C., Sun, Z.W., Ye, B.T., Liu, D.N., Zhang, L.L., Shen, L.P., Hao, Y.F., Ma, Y.Z., Lu, F. and Guo, Z.F. (2023) A high-resolution genotype-phenotype map identifies the *TaSPL17* controlling grain number and size in wheat. *Genome Biol.*, **24**, 196.

Lv, B., Nitcher, R., Han, X., Wang, S., Ni, F., Li, K., Pearce, S., Wu, J., Dubcovsky, J. and Fu, D. (2014) Characterization of *FLOWERING LOCUS T1* (*FT1*) gene in *Brachypodium* and wheat. *PLoS One*, **9**, e94171.

Shaw, L.M., Lyu, B., Turner, R., Li, C., Chen, F., Han, X., Fu, D. and Dubcovsky, J. (2019) *FLOWERING LOCUS T2* regulates spike development and fertility in temperate cereals. *J. Exp. Bot.*, **70**, 193–204.

Shaw, L.M., Turner, A.S., Herry, L., Griffiths, S. and Laurie, D.A. (2013) Mutant alleles of *Photoperiod-1* in wheat (*Triticum aestivum* L.) that confer a late flowering phenotype in long days. *PLoS One*, **8**, e79459.

VanGessel, C., Hamilton, J., Tabbita, F., Dubcovsky, J. and Pearce, S. (2022) Transcriptional signatures of wheat inflorescence development. *Sci Rep-Uk*, **12**, 17224.

Waddington, S.R., Cartwright, P.M. and Wall, P.C. (1983) A quantitative scale of spike initial and pistil development in barley and wheat. *Ann. Bot.*, **51**, 119-130.
