# Supplementary material for: LEAFY and WAPO1 jointly regulate spikelet number per spike and floret development in wheat
Source: Development. 2024 Jul 31;151(15):dev202803. doi: 10.1242/dev.202803 (PMC11317094; doi:10.1242/dev.202803)
Supplement: Supplementary file 1 [file develop-151-202803_review_history.pdf]

LEAFY and WAPO1 jointly regulate spikelet number per spike and floret development in wheat

Francine Paraiso, Huiqiong Lin, Chenxia Li, Daniel P. Woods, Tianyu Lan, Connor Tumelty, Juan M Debernardi, Anna Joe and Jorge Dubcovsky
DOI: 10.1242/dev.202803

Editor: Dominique Bergmann

Review timeline

Original submission:	18 February 2024
Editorial decision:	9 April 2024
First revision received:	3 June 2024
Accepted:	24 June 2024

Original submission

First decision letter

MS ID#: DEVELOP/2024/202803

MS TITLE: LEAFY regulates spikelet number per spike and floret development in wheat

AUTHORS: Francine Paraiso, Huiqiong Lin, Chenxia Li, Daniel P. Woods, Tianyu Lan, Juan M Debernardi, Anna Joe, and Jorge Dubcovsky

I have now received all the referees reports on the above manuscript, and have reached a decision. The referees' comments are appended below, or you can access them online: please go to BenchPress and click on the 'Manuscripts with Decisions' queue in the Author Area.

The overall evaluation is positive and we would like to publish a revised manuscript in Development, provided that the referees' comments can be satisfactorily addressed. Please attend to all of the reviewers' comments in your revised manuscript and detail them in your point-by-point response. You will notice that all three reviewers have questions about how the reported expression patterns of the genes you studied lend themselves to the models of regulation you propose, and point out areas where the discussion of data is hard to follow, and I agree that working to make the paper more accessible to readers is an important revision.

If you do not agree with any of their criticisms or suggestions explain clearly why this is so. If it would be helpful, you are welcome to contact us to discuss your revision in greater detail. Please send us a point-by-point response indicating your plans for addressing the referees' comments, and we will look over this and provide further guidance.

Reviewer 1

Advance summary and potential significance to field

In the manuscript "LEAFY regulates spikelet number per spike and floret development in wheat", the authors describe the role of LEAFY in spikelet development in wheat. To investigate the role of LEAFY in this process, they identified mutants for LFY-A and LFY-B and analysed the effect of this mutation on spikelet number per spike (SNS) and flowering time. They found that lfy shows less SNS compared to the wild type, but this phenotype is rescued when LFY-A is overexpressed in lfy under

© 2024. Published by The Company of Biologists under the terms of the Creative Commons Attribution License (https://creativecommons.org/licenses/by/4.0/).

the Ubiquitin promoter. They then investigated the molecular mechanism by which LFY controls spikelet number. To do this, they first suggested that LFY function is mediated by its interaction with WAPO1 by studying their interaction by co-immunoprecipitation, the lfy wapo1 double mutant phenotype and their expression localisation in wheat spikelets at different time points during development. They also studied the spatial localisation of several VRN1 and FUL2, which are SQUAMOSA MADS box transcription factors involved in spikelet development and the IM>TS transition. They observed that the expression of VRN1 and FUL2 in lfy is comparable to that of the wild type and they characterised their spatial localisation in the wild type. In parallel, they also studied the distribution of floral identity genes (AP3, PI1, AG1, AG2, SEP1-2, SEP1-4, SEP1-6, SEP3-1 and SEP3-2) in the wild type and described their colocalisation with LFY at W3.25 stage. Finally, they studied the genetic interaction between LFY and VRN1 and FUL2 and their effect on SNS.

I found that the authors used innovative techniques, such as single-molecule fluorescence in situ hybridisation, to carry out this research and they provide beautiful images of spikelet development in wheat. However, I find that some of the conclusions suggested by the authors are not well supported by the data and I also find that their way of presenting the data is often unclear. In particular, I find very speculative the interaction between LFY and WAPO1 in wheat and their role in SNS due to the different localisation of LFY and WAPO1 in the spikelet at W3.25 stage. I also found the conclusions about the genetic interaction between LFY and VRN1 very speculative. Therefore, I have several questions that I would like to address to the authors point by point.

Comments for the author

Major revisions:

• In the first results paragraph, line 108, the authors select two lfy alleles. I was wondering if lfy-A, which causes missplicing of the third exon, really causes a knockout phenotype. Are there important lfy domains in the third exon? Then I wondered how many LEAFY genes there are in wheat. In fact, LFY-D is also mentioned in Fig. S3. Is this a third LFY gene? Is it possible that LFY-D has a redundant function to LFY-A and LFY-B? If so, I would suggest that the authors describe this at the beginning.

• I find the description of the flower defects in lfy very complicated for the reader. From line 127 to line 136, the authors describe the phenotype and show the flower defects in Figure 1F. However, the quantification of these data is only described and no graph is shown to support the description. Indeed, the raw numbers are in data S2, but the wild type quantification of the floral organs is missing. I strongly suggest that the authors add more graphical representation of their data, this will make the paper more accessible.

• Lines 137 to 152: When they overexpress UBI:LFY and UBI:LFY-HA in wild type and lfy background, they see a rescue of the phenotype in lfy and a decrease in yield in wild type. Why does this happen? They also tested 5 independent lines for each construct, but it is unclear to me which of the 5 tested lines they chose for the following experiments? Again, the data are presented as a table in Data S5, but I will find it more accessible to the reader to produce a graph showing the data reported in Data S5. Perhaps the analysis of flower morphology in the lfy and wt overexpressor lines could add new information to the characterisation of these lines.Line 183-186: I am not sure about the conclusion that the authors reach in this paragraph. They suggest that the non-addittive effect in SNS number among lfy, wapo1 and lfy wapo1 suggest that these proteins are acting together. I will also add the possibility that these genes acts epistatically one to the other.

• Line 218 to 222: The authors study the expression level of LFY and WAPO1 at the W3.25 stage. In Figure 4, the localisation of the two proteins at this stage seems to be complementary in the spikelets, as also described in the text, which contradicts the protein-protein assay performed earlier. The authors try to address this point in the next paragraph (lines 223-233), suggesting that in more developed spikelets LFY and WAPO1 colocalise in the distal part. However, without proper quantification or measurements of the distance of WAPO1 and LFY from the tip of the spikelet meristem, it is difficult to assess whether colocalisation actually occurs. Accordingly, with this concern about LFY-WAPO1 colocalisation, I suggest that the authors re-examine their conclusion in lines 364 to 367 of the Discussion. Furthermore, in the Discussion (lines 476 to 481), the authors suggest that LFY and WAPO1 could regulate different genes when they interact or when they act

© 2024. Published by The Company of Biologists under the terms of the Creative Commons Attribution License (https://creativecommons.org/licenses/by/4.0/).

alone. However, the lfy wapo1 double mutant phenotype does not support this hypothesis. In fact, the double mutant did not show any additive effect with respect to the single mutant.

• Line 256 to 265: The authors have quantified the hybridisation signal of FUL2, VRN1 and LFY at different stages of spike development. However, it is difficult to understand which is the quantified part of the picture. Could the authors provide a schematic of the region they quantified? I think maybe this information is in data S8, but it seems very hard to understand. Also, I cannot find any information about how they count the single molecule points in the images. If possible, I will suggest to add the quantification of WAPO1 as well. Because the authors suggest that LFY promotes VRN1 and FUL expression, is it possible to study VRN1 and FUL2 localization in lfy?

• - Line 292 to 301: The authors examine the interaction between LFY and VRN1 and claim that the effect of LFY was strong in the vrn1 mutant background. Regarding these data, I disagree with the way the data are presented in Figure 6 and then with the conclusions that the authors draw. Indeed, if you go to data S12 (where the raw data are) it is possible to see that in terms of SNS lfy mutants decrease in number with respect to the wild type, but vrn1 has almost double the SNS than the wild type. Consistent with the role of lfy in reducing the number of SNS, the double lfy vrn1 has less SNS than vrn1 in a number comparable to that of the wild type. These results only confirm the importance of lfy in determining the number of SNS in the vrn1 mutant background, but provide few information about the interaction between lfy and vrn1. In fact, no epistasis of lfy on vrn1 is demonstrated, since the double mutant has a phenotype intermediate between that of the single mutants. I strongly recommend that the authors change the graphs in Figure 6 and rephrase the conclusions for this paragraph. Furthermore, if they want to give more insight into the role of LFY in the vrn1 mutant, I would suggest that they check the expression of LFY in this genetic background to see if it is upregulated.

Minor revisions:

• Lines 33 to 36: I will rephrase this sentence "Single-molecule ....SNS in wheat". I have found that this is not demonstrated in the manuscript.

• Line 162 to 173: For protein-protein interaction they use 2 different alleles of WAPO1 (WAPO-A1-47F and WAPO-A1-47C). They suggested that WAPO-A1-47F has a higher number of SNSs than WAPO-A1-47C (Kuzay et al 2022). It is unclear to me why they tested both versions. Was it because they thought this AA might be relevant for WAPO-LFY interaction? In Arabidopsis, which AA are important for this interaction? Are they known?

• Line 180: "...homologs" is misspelled.

• Line 211 to 217: The authors describe the expression of FRIZZY PANICLE (FZP) in wheat. I do not understand the rationale for this experiment. Is FZP used as a marker for a specific region of the meristem to better describe the LFY expression profile? I think the authors should better explain their intentions here.

• Line 278: I think they are referring to Figure 4D and not Figure 5D.

• Line 281 to 285: Why did they do the real time at stage W4.0 and not at stage 3.25/3.5 as in the rest of the paper?

• Lines 372 to 374: "These results.... In Arabidopsis." I disagree with this sentence because the interaction between LFY and WAPO1 is not well demonstrated and the data provided by the authors is not sufficient to support this sentence.

• Line 413 to 414: I think this sentence should be changed to make it clearer to readers.

• Line 415: I suggest changing this title.

<u>Reviewer 2</u>

© 2024. Published by The Company of Biologists under the terms of the Creative Commons Attribution License (https://creativecommons.org/licenses/by/4.0/).

Advance summary and potential significance to field

see below

Comments for the author

The present study by Paraiso et al. applied genetic, biochemical, and molecular approaches to better understand spike development in wheat and its regulation through LFY in combination with WAPO1 and other MADS-box regulators. Even though there are no major concerns related to the gene identification and interaction studies of LFY; it seems difficult for the authors to bring together the detected expression data with the found phenotype—more SNS! Considering the presented work, several issues remain and may require reconsideration or interpretation:

1) Considerations for LFY: I kind of agree with the authors that wheat LFY affects proper floral development. While this is easy to be explained by the homeotic floret mutant phenotypes (not only in wheat but also from other grasses!), mRNA expression domains in the developing spike and spikelet are less straight forward. If wheat LFY promotes floral development and growth, authors may need to explain why, during DR stage, LFY is only expressed in the leaf ridge (LR) but not in the developing spikelet ridge (SR), and how this might work? One possibility could be that wheat LFY expresses in peripheral domains of the floral meristems and acts non-cell autonomously into the above-lying floral meristem. For example, if wheat LFY mutant (lfy) plants would have a pronounced LR over the SR in the most basal DRs it could be diagnostic for floral promotion in wt plants (i.e. less LR in most basal DRs!). That means lfy plants may have similarly enhanced basal LRs as SVP mutants have (pl. see e.g. Backhaus et al. 2022 and 2023). If confirmed in a small experiment, it may suggest that wheat LFY provides short-range, non-cell autonomous signals for SM formation and growth from the LR. This would be in line with previously proposed peripheral signaling centers occurring in grass inflorescences (pl. see Whipple NP 2017). The similar idea would be applicable for the later stage expression domains (2.5 and 3.25). Here, wheat LFY expresses in the peripheral zone of the SM in a ring-like band; in an area where already differentiation most likely has occurred towards floret meristem (FM) identity. Unfortunately, authors do not provide data on the later occurring floret related expression domains of wheat LFY. However, from Selva et al 2021 it can be deduced that barley LFY shows FM and floral organ expression patterns, suggesting that wheat LFY may follow a very similar trend. In summary, I thus would encourage authors to at least discuss this above-mentioned possibility during their discussion.

2) Considerations for WAPO1 expression domains: I've had a hard time to reconcile the seen expression domains with the proposed function of IM>TS transition and more SNS. Perhaps there's something like protein transport as authors discussed; but less likely. However, an alternative idea could be that WAPO1 is involved in meristem maturation. And if so, high expression of WAPO1 in the SM (W3.25) could produce more SNS through a delayed progression towards FMs in the most matured floral primordia while in parallel expanding the time for TS transition. This might also suggest that WAPO1 and wheat LFY might work collectively during floral progression but in a highly balanced mode modulating SNS. If one of them is lacking, SNS will be low because the other partner "takes-over" and induces premature meristem maturation (wapo1<LFY = overly floral induction/promotion; or WAPO1>lfy = too low floral induction/promotion). This would also be in line with the double mutant result. Short-range signaling or protein transport from the periphery to the meristem is also in line with the found direct interactions of both proteins. Authors may want to re-assess the ubi-LFY lines and associated phenotypes for the same context. They mentioned here that these lines could only partially complement wt plants and had fertility problems, most likely due to ectopic expression and pleiotropic effects. I assume that the ubi-LFY line also needs to have an up-regulated WAPO1 to facilitate more SNS. A hypothesis to be tested, I guess.

<u>Reviewer 3</u>

Advance summary and potential significance to field

The manuscript by Paraiso et al. characterizes lfy mutants in durum wheat, also showing LFY relationship with WAPO1 and some MADS box genes. It also presents a detailed and informative transcriptional profiling of these genes during inflorescence development by smFISH.

© 2024. Published by The Company of Biologists under the terms of the Creative Commons Attribution License (https://creativecommons.org/licenses/by/4.0/).

After screening of a durum wheat mutagenized population, one LFY truncated allele for genome A and one for genome B were isolated and crossed to obtain a lfy mutant. Consistently with LFY role in other species, the mutant showed shorter spikes bringing less spikelets. Interestingly, a dosage effect was also observed with A and B mutants showing intermediate phenotypes. Additionally, lfy mutants showed florets with sovra-numeral organs and mixed identities probably caused by altered expression of MADS box floral identity genes. LFY overexpression under a constitutive promoter could largely revert the lfy mutant defects, confirming LFY as the causal gene.

Since LFY interacts with APO1/UFO in other species and similar phenotypes for the mutants were observed, this interaction was also shown in durum wheat by co-IP. smFISH was widely used in wt tissues to study the localization of LFY, WAPO1 and also MADS BOX genes in a temporal manner while expression of some of these genes was also shown deregulated by qRT in lfy mutant smFISH could describe at high-definition and also in a semi quantitative manner the expression dynamics of quite a number of floral identity MADS box genes during development and their dependency on LFY was also suggested. Although at different level, vrn1 and ful2 mutations exacerbated the SNS decrease observed in lfy mutant, showing genetic interaction between these genes, especially for lfy and vrn1. LFY in wheat shows therefore some similarities with Arabidopsis but also many differences, that are shared with other grasses.

Overall, the manuscript is very interesting for the field and discusses important similarities between Arabidopsis and grasses and in particular wheat that help understanding molecular pathways that control the development of differeny types of inflorescences.

Comments for the author

1)TITLE: I would try to mention the MADS box also in the title, in relation to LFY, as much information on their function is in the manuscript too.

2) Since the mutation in the B genome is quite at the end of the cds and LFY overexpressing lines in lfy background could not fully complement, other alleles or RNAi would remove any doubt. I understand nevertheless that it might not be easy and especially not fast to do it.

3) Concerning LFY/WAPO interaction:
smFISH show very nicely where molecules of LFY or WAPO1 mRNA are found: although it is proposed that the two proteins work together, I am not sure that their expression largely overlaps. It looks that only few cells show both transcripts, and only in specific developmental stages. Do you think that the proteins might move?
-Although WAPO-LFY interaction was shown in other species and it was not surprising, in wheat too it could be confirmed by another method other than co-IP. Also: how is the complex formed? Is it a dimer or a tetramer? Could you conclude something on that? LFY-WAPO interaction was tested using two allelic variants of WAPO but which is Kronos's variant?

4)Which wapo1 mutants were used in this experiment? Which are the alleles? (please specify in line 175).

5)how do wapo1 florets look like? Are they similar to lfy? Is there an additive effect between the two mutations more prominent that for SNS, that, although significant, is very low?

6) Concerning VRN1 and FUL it seems that their expression also only partially overlaps with LFY and WAPO, this also implies some protein movement or some cell specificity of MADS activation by LFY/WAPO

7) I would move the model in fig S11 (maybe simplified) to the main text…

8) LINE 189 please specify what variety is CS

9) LINE 211-217: I do not see the point of adding here FZP, it is not introduced, and it is not useful to understand LFY's function in my opinion.

© 2024. Published by The Company of Biologists under the terms of the Creative Commons Attribution License (https://creativecommons.org/licenses/by/4.0/).

10) Why in figure 1C the experiment was done on 9 plants while in FigS1 on 27? Are they two independent experiments?

First revision

Author response to reviewers' comments

Answers to reviewers

Author answer to all reviewers and new results: All three reviewers expressed concerns regarding the lack of co-localization of the expression of *WAPO1* and *LFY* in the IM at the time of the IM→TS transition, and this was an area we were also doing more work to clarify. The similar number of spikelets per spike (in wheat) or per panicle (in rice) in the individual *lfy* and *wapo1* mutants and in the combined *lfy wapo1* mutant indicates that these two genes act cooperatively in the determination of this trait.

Our initial expectation of co-localization of *LFY* and *WAPO1* expression in the inflorescence meristem was based on the hypothesis that the differences in SNS were originated by a difference in the timing of the IM→TS transition. However, our latest experiments demonstrate that this is not the case, and that the reduced SNS in the *lfy* and *wapo1* mutants is caused by a slower rate of spikelet meristem (SM) formation relative to the wildtype (rate = SM/d) rather than by a different timing in the IM→TS transition. This reduced rate of spikelet meristem formation was evident from the beginning of the spike development, a stage when *LFY* and *WAPO1* expression is colocalized in the IM!

We added two new experiments as figures 3C and 3D, describing the changes in spikelet meristem formation with time, both showing a drastic reduction in the rate of spikelet formation per day (sm/d) from the initial stages of spike development. Moreover, the second experiment demonstrated that the reduction in sm/d relative to the wildtype is the same for the two mutants, indicating that LFY and WAPO1 jointly regulate this rate.

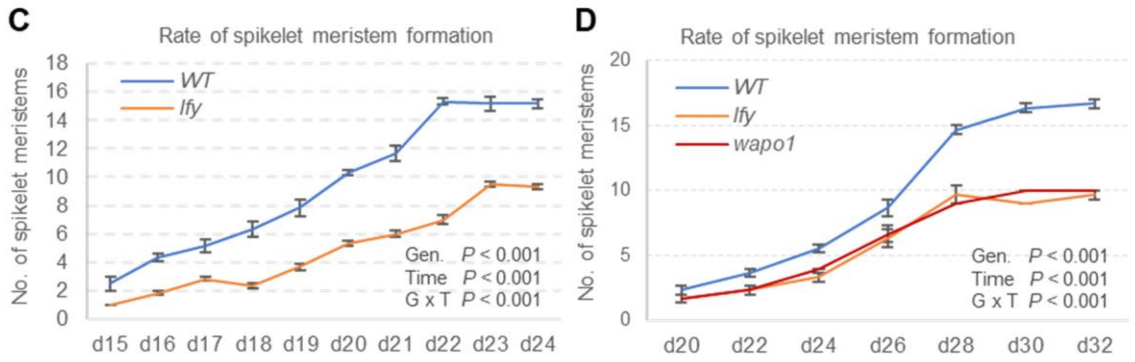

Fig. 3. (**C-D**) Changes in the number of spikelet meristems with time. Numbers after the letter d indicate days from germination (**C**) Wild type Kronos and *lfy* mutant grown under long days (16 h light, n = 6 per genotype - time point) (**D**) Wild type Kronos, *lfy*, and *wapo1* mutants grown first under short day (8 h light) for eight days and then transferred to long days (n = 3 per genotype - time point). *P* values correspond to a repeated measures analysis. A significant genotype x time interactions indicates significant different rates of spikelet meristem formation. Raw data and statistical analyses are included in data S6.

We described the new results in the following paragraph: "To test if the *lfy* reduction in SNS was due to a premature IM→TS transition or a reduced rate of SM formation, we dissected developing spikes and recorded the variation in SM number per day (sm/d). The first experiment (long days), showed a similar IM→TS transition time but a significantly faster rate of spikelet formation in the wildtype (1.83 sm/d) than in the *lfy* mutant (0.86 sm/d) (Fig. 3C, data S6). In the second experiment, we grew the seedlings for 8 days under short days and then transferred them to long

© 2024. Published by The Company of Biologists under the terms of the Creative Commons Attribution License (https://creativecommons.org/licenses/by/4.0/).

days to synchronize the reproductive transition. In this experiment, the rate of spikelet meristem formation in the wildtype (1.40 sm/d) was also faster than in the *lfy* (0.73 sm/d) and *wapo1* (0.83 sm/d, Fig. 3D). In both experiments, different rates of SM formation were observed from the earliest stages of spike development (Fig. 3C-D). Repeated measures analyses revealed highly significant differences between mutant and wildtype genotypes, time points, and genotype x time interactions, which indicates a differential response in time (data S6). There was no significant difference in the rate of SM formation when comparing the *lfy* and *wapo1* mutants alone (data S6)."

From these results it is clear that both ***WAPO1* and *LFY* affect the rate of formation of spikelet meristems** rather than the timing of the IM→TS transition, and **that these changes are evident from the initial stages of spikelet development, when *LFY* and *WAPO1* are co-expressed in the IM** (Fig. 4A).

Based on our improved understanding of the way *LFY* and *WAPO1* control SNS, we modified the discussion section. We eliminated the previous speculations and presented a simpler hypothesis: "*LFY* was co-expressed with *WAPO1* in the IM at the early stages of spike development (Fig. 4A) but not at later stages (Fig. 4B-C and Fig. S5B-C). This early co-localization in the IM seems to be sufficient to explain the similar reduction in SNS observed in the *lfy* and *wapo1* mutants. Both mutants were associated with similar reductions in the rate of SM formation relative to the wildtype, rather than by a change in the timing of the IM→TS transition (Fig. 3C-D). Since these differences were evident from the earliest stages of spikelet development, when both *LFY* and *WAPO1* were co-expressed in the IM (Fig. 4A), we hypothesize that the transient formation of the LFY-WAPO1 complex is sufficient to activate gene expression networks that accelerate the rate of SM initiation."

On the light of the new results, it is no longer critical to have LFY and WAPO1 colocalized in the IM at the time of the transition to a terminal spikelet, because the mutants and the wildtype do not differ in the timing of this this transition. Thank you for leading us to explore this question in more detail!

Reviewer 1

Advance Summary and Potential Significance to Field

In the manuscript "LEAFY regulates spikelet number per spike and floret development in wheat", the authors describe the role of LEAFY in spikelet development in wheat. To investigate the role of LEAFY in this process, they identified mutants for LFY-A and LFY-B and analysed the effect of this mutation on spikelet number per spike (SNS) and flowering time. They found that lfy shows less SNS compared to the wild type, but this phenotype is rescued when LFY-A is overexpressed in lfy under the Ubiquitin promoter. They then investigated the molecular mechanism by which LFY controls spikelet number. To do this, they first suggested that LFY function is mediated by its interaction with WAPO1 by studying their interaction by co-immunoprecipitation, the lfy wapo1 double mutant phenotype and their expression localisation in wheat spikelets at different time points during development. They also studied the spatial localisation of several VRN1 and FUL2, which are SQUAMOSA MADS box transcription factors involved in spikelet development and the IM>TS transition. They observed that the expression of VRN1 and FUL2 in lfy is comparable to that of the wild type and they characterised their spatial localisation in the wild type. In parallel, they also studied the distribution of floral identity genes (AP3, PI1, AG1, AG2, SEP1-2, SEP1-4, SEP1-6, SEP3-1 and SEP3-2) in the wild type and described their colocalisation with LFY at W3.25 stage. Finally, they studied the genetic interaction between LFY and VRN1 and FUL2 and their effect on SNS. I found that the authors used innovative techniques, such as single-molecule fluorescence in situ hybridisation, to carry out this research and they provide beautiful images of spikelet development in wheat. However, I find that some of the conclusions suggested by the authors are not well supported by the data and I also find that their way of presenting the data is often unclear. In particular, I find very speculative the interaction between LFY and WAPO1 in wheat and their role in SNS due to the different localisation of LFY and WAPO1 in the spikelet at W3.25 stage. I also found the conclusions about the genetic interaction between LFY and VRN1 very speculative. Therefore, I have several questions that I would like to address to the authors point by point.

© 2024. Published by The Company of Biologists under the terms of the Creative Commons Attribution License (https://creativecommons.org/licenses/by/4.0/).

Comments for the Author

Major revisions

1. In the first results paragraph, line 108, the authors select two lfy alleles. I was wondering if lfy-A, which causes missplicing of the third exon, really causes a knockout phenotype. Are there important lfy domains in the third exon? Then I wondered how many LEAFY genes there are in wheat. In fact, LFY-D is also mentioned in Fig. S3. Is this a third LFY gene? Is it possible that LFY-D has a redundant function to LFY-A and LFY-B? If so, I would suggest that the authors describe this at the beginning.

Author answer: In wheat there is one copy of *LFY* **per genome**, so two copies in tetraploid wheat (*LFY- A* and *LFY-B*) and three copies in hexaploid wheat (the previous two plus *LFY-D*). Since we are doing all our work in tetraploid wheat, the variation in *LFY-D* is not relevant to this study. *LFY-D* is likely to play a redundant role with the two other homeologs in hexaploid wheat, but this was not investigated in this study.

Regarding the effect of the *lfy-A* splicing mutation, we cannot rule out some small residual effects but both the sequence analyses and the observed phenotypes suggest that the selected mutations are very severe, and likely full-knockouts.

Sequence analysis: We sequenced the cDNA produced by the *lfy-A* mis-splicing mutant and found that it results in a premature stop codon that eliminates 121 amino acids in the C-terminal part of the protein, or 31% of the total amino acids. Of these 121 truncated amino acids, 105 are highly conserved across grasses and even dicot species. The position of the *lfy-A* and *lfy-B* truncations in the Kronos mutants are indicated in the figure below obtained from Rieu et al. 2023 (Nature Plants 9:315-329). This figure shows the alignment of LFY DNA binding domain (DBD) which are highly conserved from Algae to Angiosperms. Since both the *lfy-A* and *lfy-B* mutations eliminate most of the conserved DBD, it is safe to conclude that the truncated Kronos lfy-A and lfy-B proteins can no longer bind to their target DNAs and are no longer functional.

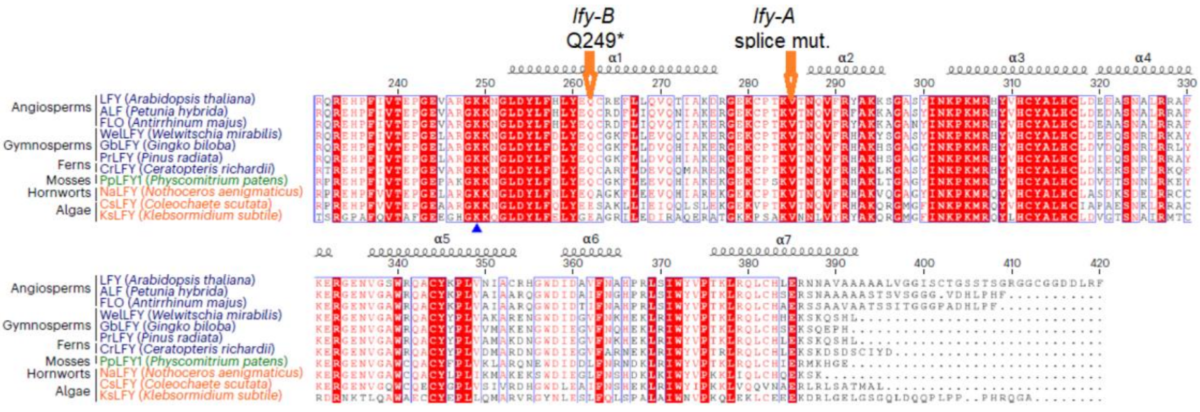

We included this information in the description of the mutants in the first section of results: "The eliminated amino acids in the two wheat mutants include the highly conserved LFY DNA binding domains, suggesting that the truncated proteins can no longer bind their target DNAs and are, most likely, not functional (Maizel *et al.*, 2005, Rieu *et al.*, 2023b) (Fig. 1A)."

Phenotypic analyses: the phenotypes of the combined *lfy-A lfy-B* mutations also suggest that the encoded truncated proteins are no longer functional. Whereas each of the mutations alone has very limited effect on SNS, the combined *lfy-A lfy-B* reduced the spikelet number per spike (SNS) to almost half, and the floral defects were so severe that the plants were mostly sterile. Since *LFY* and WAPO1 work together, a comparison of the effects of the combined *lfy-A lfy-B* and *wapo-A1 wapo-B1* mutants is also informative. The CRISPR induced premature stop codons in *wapo-A1 wapo-B1* eliminate more than 60% of the encoded proteins. We observed that the reduction in SNS was the same in both the *lfy* and *wapo1* combined mutants, suggesting that both encode similar loss-of-

© 2024. Published by The Company of Biologists under the terms of the Creative Commons Attribution License (https://creativecommons.org/licenses/by/4.0/).

function truncated proteins. The floral defects of the combined *lfy-A lfy-B* are even more severe than those of *wapo-A1 wapo-B1* suggesting that both *lfy-A* and *lfy-b* are loss-of-function mutations.

Taken together, these results indicate that the selected *lfy-A* and *lfy-B* truncations are very strong mutation, and most likely encode non-functional proteins.

2. *I find the description of the flower defects in lfy very complicated for the reader. From line 127 to line 136, the authors describe the phenotype and show the flower defects in Figure 1F. However, the quantification of these data is only described and no graph is shown to support the description. Indeed, the raw numbers are in data S2, but the wild type quantification of the floral organs is missing. I strongly suggest that the authors add more graphical representation of their data, this will make the paper more accessible.*

Author answer: As requested by reviewer #1 we added two graphs in Figure S1 summarizing the frequency of abnormal and normal floral organs in the *lfy* mutant in different parts of the spike.

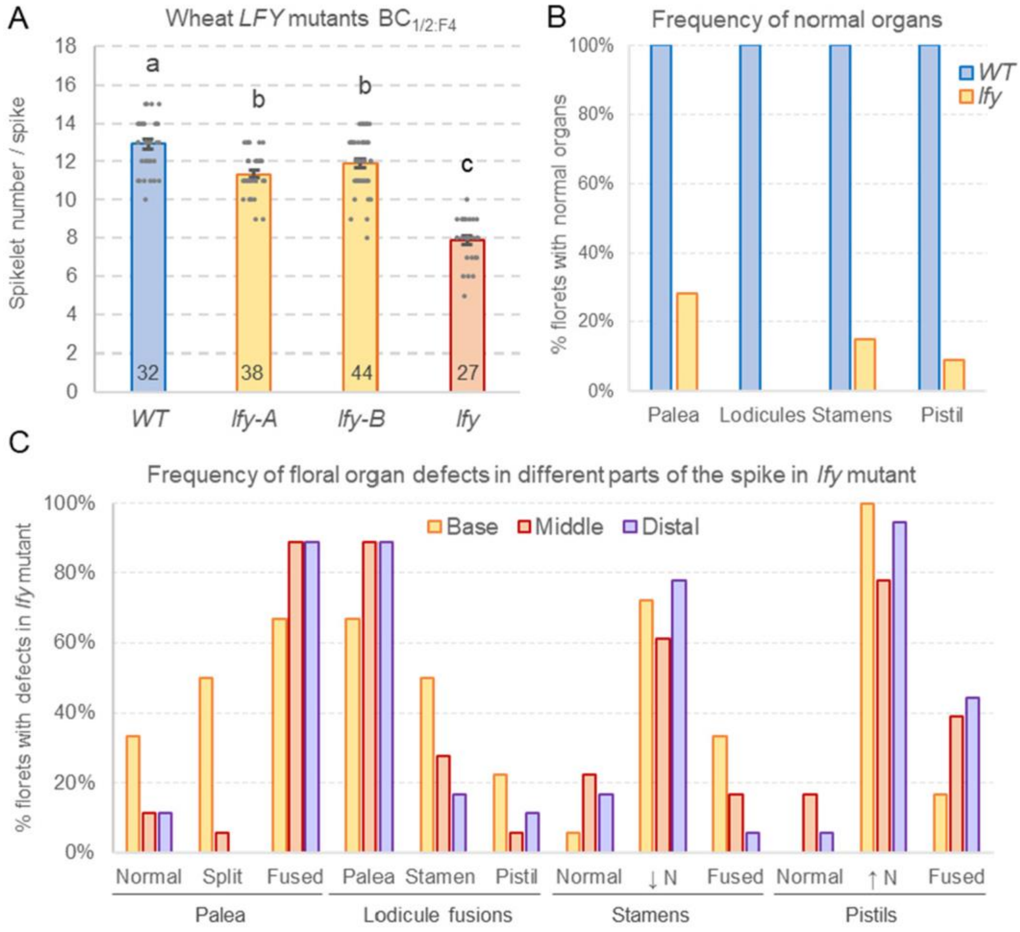

3. *Lines 137 to 152: When they overexpress UBI:LFY and UBI:LFY-HA in wild type and lfy background, they see a rescue of the phenotype in lfy and a decrease in yield in wild type. Why does this happen?*

Author answer: We are overexpressing *LFY* with a strong constitutive promoter that expresses *LFY* in all tissues and developmental phases, and at levels that are not observed in nature, so negative pleiotropic effects are expected. We added Fig. S2B and C to show the floral defects associated with the *UBI:LFY- HA* and their frequency. We added the following paragraph to this result section: "Among 14 dissected florets, we observed missing or fused lodicules in 21%, fused stamen filaments in 43% and pistils with extra stigmas in 36% of the florets (Fig. S2B-C). Floral organ defects were less frequent and less severe than in *lfy*, which explains the higher fertility of the *UBI:LFY-HA*

© 2024. Published by The Company of Biologists under the terms of the Creative Commons Attribution License (https://creativecommons.org/licenses/by/4.0/).

plants (23±10 grains / plant) relative to *lfy* (2.7±0.7 grains per plant), but its reduced fertility relative to the wildtype (94±25 grains per plant, *P*=0.019, data S5). These results indicate that the ectopic expression of *LFY* is associated with negative pleiotropic effects on floral organ development and fertility".

4. *They also tested 5 independent lines for each construct, but it is unclear to me which of the 5 tested lines they chose for the following experiments? Again, the data are presented as a table in Data S5, but I will find it more accessible to the reader to produce a graph showing the data reported in Data S5. Perhaps the analysis of flower morphology in the lfy and wt overexpressor lines could add new information to the characterisation of these lines.*

Author answer: The expression data for the transgenic plants is presented in Figure S2 and the phenotypic effect of the *UBI:LFY-HA* transgene on SNS is presented in Figure 2. Grain set is presented only as a number in the text and in data S5. Since this is a simple percentage, we did not present it in a graph. WE added Fig. S2B-C to describe the floral defects in the *UBI:LFY-HA* transgene.

The crosses between the *UBI:LFY-HA* and the *lfy* mutant were performed using transgenic plant #4, which showed intermediate levels of *LFY* expression in the leaves (data S4). The plants were self-pollinated and, in the progeny, we selected plants for the four possible classes: wildtype plants with and without the transgene, and *lfy* mutants with and without the transgene. We clarified in the text that transgenic plant #4 was used: "We then crossed the *UBI:LFY-HA* transgenic plant #4 with the *lfy* mutant, and in the progeny selected sister lines homozygous for combined *lfy* mutations or for wildtype alleles (WT), each with or without the transgenes."

While rechecking the plants used in this experiment, we discovered that both transgenic experiments were done with the *UBI:LFY-HA* transgene, so we corrected that information in the text and data S5.

5. *Line 183-186: I am not sure about the conclusion that the authors reach in this paragraph. They suggest that the non-addittive effect in SNS number among lfy, wapo1 and lfy wapo1 suggest that these proteins are acting together. I will also add the possibility that these genes acts epistatically one to the other.*

Author answer: We rephrased the sentence to indicate that *LFY* and *WAPO1* show a significant reciprocal recessive epistatic interaction: "The genetic epistatic interaction for SNS was highly significant in a factorial ANOVA, and the contrasts for the simple effects showed no-significant differences in SNS for *LFY* or *WAPO1* in the presence of the mutant allele of the other gene (data S6). These results indicate a reciprocal recessive epistatic interaction between these two genes, and that LFY and WAPO1 need each other to regulate SNS."

6. *Line 218 to 222: The authors study the expression level of LFY and WAPO1 at the W3.25 stage. In Figure 4, the localisation of the two proteins at this stage seems to be complementary in the spikelets, as also described in the text, which contradicts the protein-protein assay performed earlier. The authors try to address this point in the next paragraph (lines 223-233), suggesting that in more developed spikelets LFY and WAPO1 colocalise in the distal part. However, without proper quantification or measurements of the distance of WAPO1 and LFY from the tip of the spikelet meristem, it is difficult to assess whether colocalisation actually occurs.*

Author answer: We agree with the reviewer that claiming overlapping expression areas from *in situ* hybridization experiments using different tissue sections requires measurements to a common reference point. However, smFISH permits visualization of the two genes in the <u>same spikelet section</u>, simultaneously, and the simultaneous visualization of cell wall boundaries using calcofluor (see below).

© 2024. Published by The Company of Biologists under the terms of the Creative Commons Attribution License (https://creativecommons.org/licenses/by/4.0/).

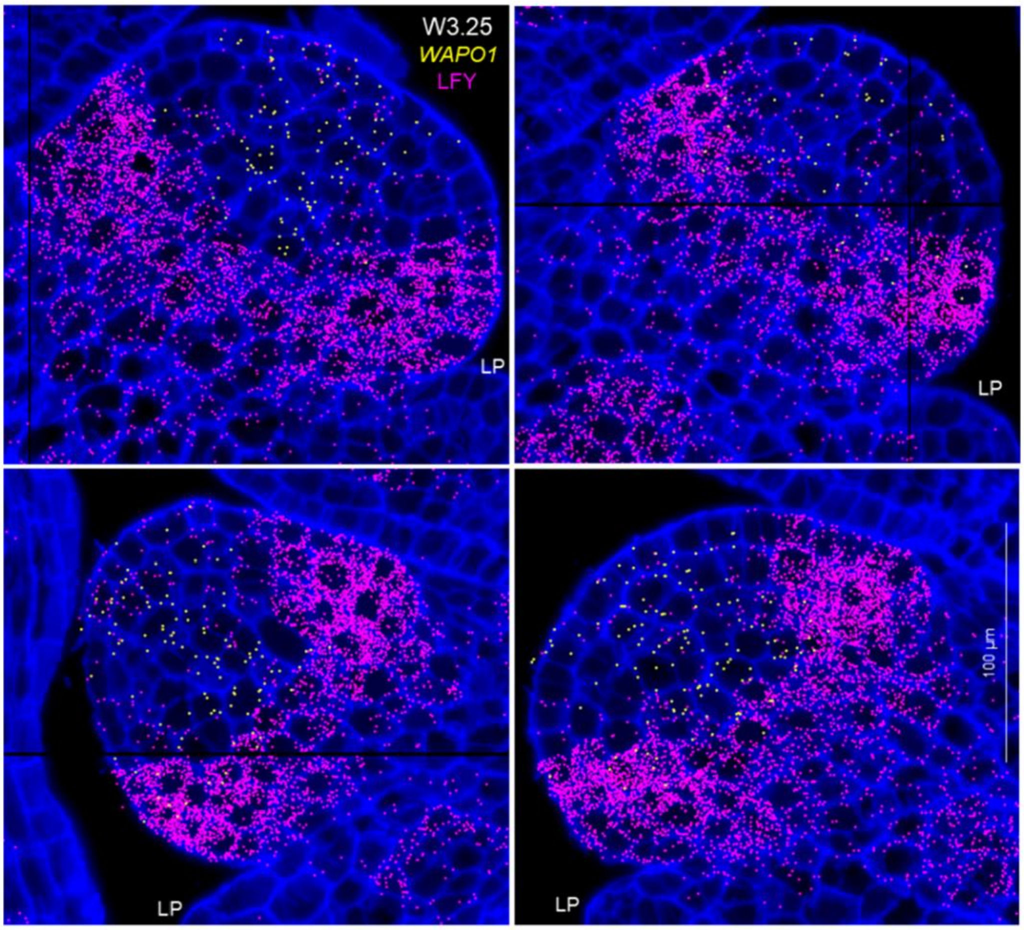

We included a new supplemental Fig. S6 showing in more detail the overlap between the expression profiles of *LFY* and *WAPO1* in four spikelet meristems at the lemma primordia (LP) stage. These smFISH pictures show that *LFY* and *WAPO1* are co-expressed in the spikelet/floral meristem distal region and that *WAPO1*expression extends to one - two cell layers deep into the area of high *LFY* expression.

This observation was consistent across multiple spikelets in different sections (Fig. S6). We hypothesize that the co-expression of *LFY* and *WAPO1* in these cells enhances the probability of interactions between their encoded proteins, and that this overlapping region provides essential spatial information for the correct expression of the floral organ identity genes and the correct flower development.

The *lfy* and *wapo1* mutants show very similar alterations in the expression of the floral organ identity genes and similar floral abnormalities. This result indicates that the presence of both proteins is required to provide adequate spatial information for floral development. The frequent organ fusions involving paleas, lodicules, anthers and pistils, suggest incomplete spatial determination of the boundaries among floral organs. We describe this new figure in the results and discussion section:

Result section: "At the lemma primordia stage (W3.25), *WAPO1* expression was also detected in the distal part of the more developed spikelets (Fig. 4D and S5C), in agreement with previous *in situ* hybridization results (Kuzay *et al*. 2022). A detail of the *WAPO1* expression domain shows co-localization with *LFY* in multiple cells within the distal region of the developing spikelet, which extends to one - two cell layers into the area of high *LFY* expression (Fig. S6)."

Discussion section: "Within the distal region of the developing spikelets, *WAPO1* was co-expressed with *LFY* in multiple cells, including one or two cell layers within the region of high *LFY* expression (Fig. 4D and S6). Within this overlapping region LFY and WAPO1 proteins may have a higher chance to interact with each other, providing valuable spatial information to the floral organ identity

© 2024. Published by The Company of Biologists under the terms of the Creative Commons Attribution License (https://creativecommons.org/licenses/by/4.0/).

genes. This hypothesis is indirectly supported by similar reductions in the expression levels of the floral organ identity genes and similar floral abnormalities in both the *wapo1* and *lfy* mutants (Fig. 1F, S11, (Kuzay *et al.*, 2022)).

7. Accordingly, with this concern about LFY-WAPO1 colocalisation, I suggest that the authors re-examine their conclusion in lines 364 to 367 of the Discussion. Furthermore, in the Discussion (lines 476 to 481), the authors suggest that LFY and WAPO1 could regulate different genes when they interact or when they act alone.

Author: The new Fig S6 provides good evidence of the *LFY* and *WAPO1* colocalization within the same cells. This spatial and temporal co-localization overlaps with the expression domains of the floral organ identity genes (Fig. 5E-F). In Arabidopsis, it has been shown that the UFO-LFY complex binds to the promoters of the floral organ identity genes *AP3* and *PI* directly regulating their expression.

To link better our results with the published results in Arabidopsis, we added the following paragraph: "In Arabidopsis, the UFO-LFY complex regulates a different set of gene targets than LFY alone (Rieu *et al.*, 2023b), and both genes are required for the correct regulation of the floral organ identity genes *AP3* (Chae *et al.*, 2008, Rieu *et al.*, 2023b) and *PI* (Honma and Goto, 2000). These results are consistent with the down-regulation of the wheat floral organ identity genes in the *lfy* and *wapo1* mutants, and with the overlap between the expression domains of the wheat floral organ identity genes (Fig. 5 and S10) and *LFY* - *WAPO1* co-expression in the distal part of the wheat developing spikelets."

8. However, the lfy wapo1 double mutant phenotype does not support this hypothesis. In fact, the double mutant did not show any additive effect with respect to the single mutant.

Author: Since these two genes show reciprocal recessive epistatic interactions, the expected result is an identical phenotype in the double and single mutants. This type of epistasis is frequently associated with two genes encoding peptides that are part of a protein complex, and that are both essential for its function. Therefore, the physical interaction between LFY and WAPO1 confirmed by coIP and the reciprocal recessive epistatic interaction demonstrated by the genetic experiments are consistent with the observed effects of the mutants on SNS.

9A. Line 256 to 265: The authors have quantified the hybridisation signal of FUL2, VRN1 and LFY at different stages of spike development. However, it is difficult to understand which is the quantified part of the picture. Could the authors provide a schematic of the region they quantified? I think maybe this information is in data S8, but it seems very hard to understand. Also, I cannot find any information about how they count the single molecule points in the images.

Author: When the IM starts its transition to a terminal spikelet, the immediate lateral meristems will develop into glumes rather forming new spikelets, as demonstrated by the appearance of *FZP*. Therefore, it is important to quantify the expression changes in both the IM and the two immediate lateral meristems during this transition. In supplemental data S8, we provide the exact areas used to measure hybridization signal intensity in each distal region using ImageJ and Polylux v1.9. The IM area is marked in yellow and the area of the first and second lateral meristems in green and red, respectively.

© 2024. Published by The Company of Biologists under the terms of the Creative Commons Attribution License (https://creativecommons.org/licenses/by/4.0/).

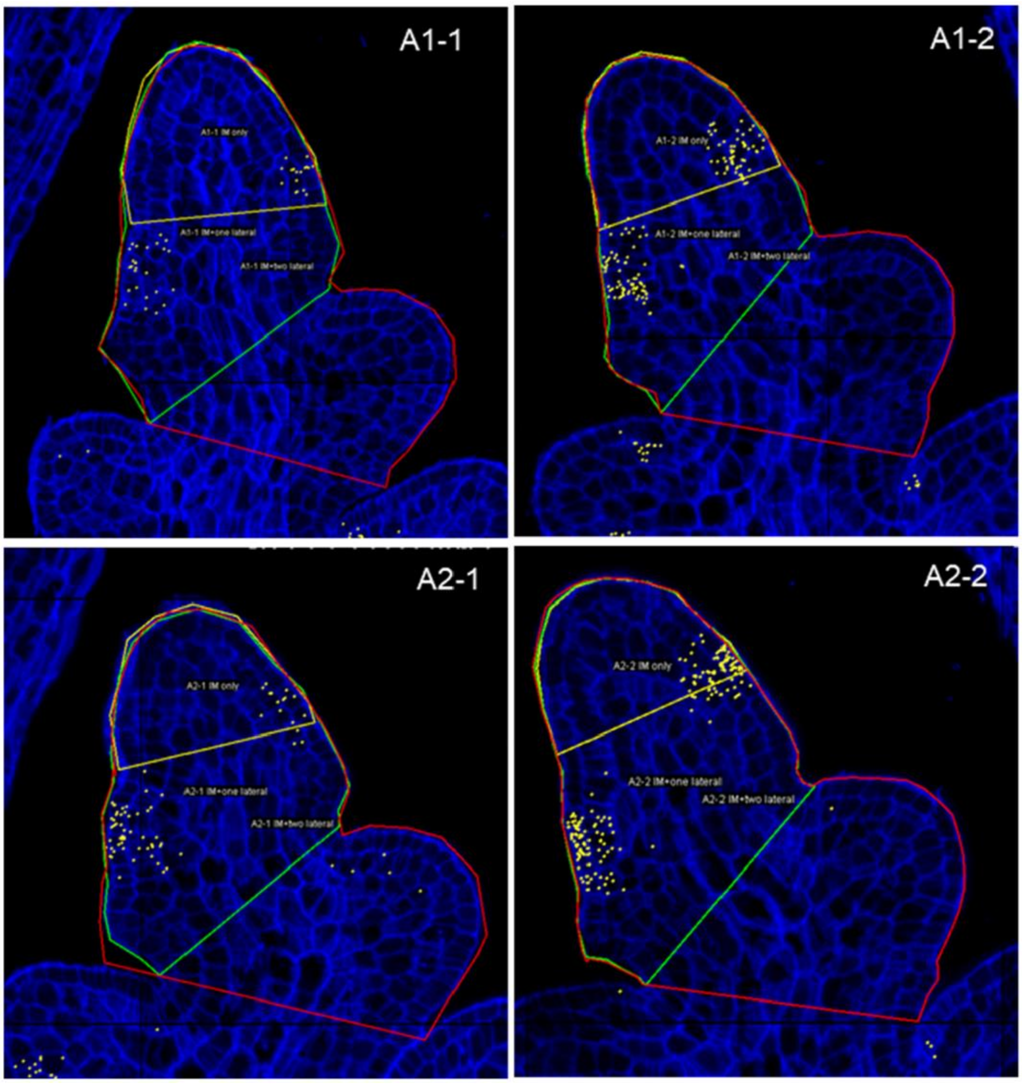

We provide the measurements for both the IM alone and for the complete terminal region that will become the terminal spikelet, which include the first two lateral meristems expressing *FZP*. Both results are very similar and support each other. We clarified the meaning of the colored areas in figures and the rationale for measuring both areas in the legend of data S8:

"In the figures presented in this spreadsheet, we delimited the regions used to quantify hybridization signal density (dots /100 µm2) using a yellow line to delineate the IM area and green and red lines to delineate the first and second lateral meristems, respectively. These lateral meristems are part of the new terminal spikelet at W3.25.

As an additional control, we quantified the hybridization signal of each target gene relative to the hybridization signal of the cell division gene CDC20, which showed a uniform distribution in the spike. Finally, we compared the ratios of SQUAMOSA / LFY in each region measured. These ratios are independent of the normalization method used.

Imaging and image processing was performed as described before (Glenn et al., Theor. Appl. Genet, 2023:136-237). Dot density was determined using ImageJ and Polylux v1.9".

9. *B. If possible, I will suggest to add the quantification of WAPO1 as well.*

WAPO1 hybridization signal was not quantified because it is not express in the terminal part of the spike at W3.0 or W3.25 (see Fig. S5)

© 2024. Published by The Company of Biologists under the terms of the Creative Commons Attribution License (https://creativecommons.org/licenses/by/4.0/).

9.C. Because the authors suggest that LFY promotes VRN1 and FUL expression, is it possible to study VRN1 and FUL2 localization in lfy?

In Arabidopsis and several eudicots, *LFY* operates as a direct promoter of the SQUAMOSA MADS-box genes *AP1* and *CAL*, but that is not the case in wheat and other grasses. Our qRT-PCR results in Figures S7A and S7B (at W2.0 and at W3.0 stage) failed to reveal any significant differences in the expression of *VRN1* or *FUL2* between the wildtype and combined *lfy* mutant.

However, we agree with the reviewer that it will be very interesting to perform an additional spatial transcriptomics study including the *lfy* and *wapo1*. This will be particularly informative to see the effects of the absence of these two genes on the expression domains of the floral organ identity genes.
Unfortunately, spatial transcriptomic experiments are very expensive ($25K) and we were not able to accommodate everything in it.

10. Line 292 to 301: The authors examine the interaction between LFY and VRN1 and claim that the effect of LFY was strong in the vrn1 mutant background. Regarding these data, I disagree with the way the data are presented in Figure 6 and then with the conclusions that the authors draw. Indeed, if you go to data S12 (where the raw data are) it is possible to see that in terms of SNS lfy mutants decrease in number with respect to the wild type, but vrn1 has almost double the SNS than the wild type. Consistent with the role of lfy in reducing the number of SNS, the double lfy vrn1 has less SNS than vrn1 in a number comparable to that of the wild type. These results only confirm the importance of lfy in determining the number of SNS in the vrn1 mutant background, but provide few information about the interaction between lfy and vrn1. In fact, no epistasis of lfy on vrn1 is demonstrated, since the double mutant has a phenotype intermediate between that of the single mutants. I strongly recommend that the authors change the graphs in Figure 6 and rephrase the conclusions for this paragraph. Furthermore, if they want to give more insight into the role of LFY in the vrn1 mutant, I would suggest that they check the expression of LFY in this genetic background to see if it is upregulated.

Author: We agree with the reviewer that the effects of *lfy* and *vrn1* are “mostly” additive. In the presence of the WT allele of the other gene, the *vrn1* mutant adds 9.2 spikelets and the *lfy* mutant reduces 5.2 spikelets. Therefore, a purely additive model would have resulted in a double mutant with 13.4 + 9.2 -5.2 = 17.4 spikelets, instead of the 12.2 observed. The difference between the expected and observed phenotypes of the double mutants 17.4 - 12.2 = 5.2 spikelets is the interaction effect. The statistical analysis using a factorial ANOVA demonstrates that these *LFY* x *VRN1* interaction effect for SNS is highly significant (P<0.0001) as calculated in data S12.

We recently added an additional factorial experiment with a larger number of replications to test the *LFY* x *FUL2* interaction. This interaction effect (2.0 spikelets) is smaller than the *LFY x VRN1* interaction effect, but in the larger experiment it was also significant (P= 0.0175). The significance of the *LFY x FUL2* interaction reinforces the previous results and suggest a more general *LFY x SQUAMOSA* interaction. In addition, the simple effects in the interactions are in the same direction: the effect of the *LFY* mutants is larger in the absence of the functional *SQUAMOSA* gene, whereas the effect of both *SQUAMOSA* genes is larger in the presence of the wildtype *Lfy* allele. Taken together, these results provide very strong experimental evidence for the existence of a *LFY x SQUAMOSA* interaction for SNS. Since epistasis is defined as the significant interaction of two genes on a trait, these results demonstrate a significant epistatic interaction between *VRN1* and *LFY* for SNS.

However, the proportion of variation explained by these interactions is less than one fifth of the total variation, so it is difficult to perceive it without statistical analyses. Even though the proportion of explained variation by this interaction is small relative to the additive effects, it is still important to report its existence to inform future molecular experiments and to guide breeders to select alleles in the different genes that maximize SNS. In addition, this interaction is important to understand the biology behind the differences in SNS. In our unpublished RNAseq studies, we identified 126 differentially expressed genes (DEG) regulated by both *LFY* and *VRN1/FUL2*, and 99 of them were regulated in opposite directions. We hypothesize that these common targets may contribute to the observed genetic interaction reported in this study, which opens interesting

© 2024. Published by The Company of Biologists under the terms of the Creative Commons Attribution License (https://creativecommons.org/licenses/by/4.0/).

biological questions.

The reviewer indicates that the double *vrn1 lfy* mutant is intermediate to the single mutants, but that is not the case. The difference between the double mutant and *lfy* alone is 4.02 spikelets whereas the difference between the double mutant and *vrn1* alone is 10.4 spikelets (more than double). This highlights the need of specialized graphs to visualize interactions. The interaction graphs allow readers to visualize interactions as a departure from parallel lines, which represent 100% additive effects. For example, a rapid analysis of Fig. 6, reveals non-parallel lines in 6A (SNS, significant interaction) and parallel lines for 6B (leaf number) and 6C (heading time), which both show non-significant interactions. Since not every reader is familiar with interaction graphs and their interpretation, we added that information in the legend of Fig. 6: "In the interaction graphs, parallel lines indicate additive effects and non-parallel lines reflect interactions."

Minor revisions:

•Lines 33 to 36: I will rephrase this sentence "Single-molecule SNS in wheat". I have found that this is not demonstrated in the manuscript.

Author response: We agree with the reviewers that we cannot compare total smFISH intensities among different genes because they depend on the hybridization strength of the designed probes. However, we are not making comparisons among genes. We are comparing the same gene across two developmental stages (W2.5 and W3.25). We performed these comparisons using two separate normalization methods (dot density and relative dot density to an internal control) and both generated the same result. Moreover, the critical comparison presented in this study is the change in the ratio between *SQUAMOSA* and *LFY* hybridization signal. Since this is a ratio of two genes in the same sections, we are also correcting for any potential section effects. The observed changes in the SQUAMOSA/LFY ratios between W2.5 to W3.25 were not small: the *VRN1/LFY* ratio in the IM increased **8-fold** between the two stages and the *FUL2/LFY* ratio increased **25-fold** between the two stages (data S8), and both increases were statistically significant (n = 4 sections at each stage). In addition, the differences are significant both when the IM is considered separately or when it is combined with the two closest lateral meristems that will be also part of the terminal spikelet. We added the following sentence to the discussion section: "In the IM, the *VRN1/LFY* ratio increased more than 8-fold and the *FUL2/LFY* ratio increased more than 25-fold between W2.5 and W3.25 at the initiation of the terminal spikelet (Fig. S87 and data S8)."

Moreover, a reduction of *LFY* expression is also evident in the spikelet meristems from the earlier stages of spikelet development (Fig.4B and 4C), supporting the idea that a reduction in *LFY* expression is required for the specification of spikelet meristem identity both during the IM→TS transition and in the lateral meristems.

•Line 162 to 173: For protein-protein interaction they use 2 different alleles of WAPO1 (WAPO-A1-47F and WAPO-A1-47C). They suggested that WAPO-A1-47F has a higher number of SNSs than WAPO-A1-47C (Kuzay et al 2022). It is unclear to me why they tested both versions. Was it because they thought this AA might be relevant for WAPO-LFY interaction? In Arabidopsis, which AA are important for this interaction? Are they known?

Author response: We clarified that we tested the interaction of LFY with both WAPO-A1 alleles to see if this polymorphism affected the interaction: "We used co-immunoprecipitation (Co-IP) to test the interaction between LFY-A and two WAPO-A1 natural alleles that differ in the presence of a cysteine or a phenylalanine at position 47 to see if this polymorphism affects the interaction." The C47F polymorphisms in wheat is within the conserved F-box domain which is not required for UFO interactions with LFY in Arabidopsis (Chae et al. 2008). However, since there are reported differences in the LFY-UFO binding regions among different species, and we could not rule out an indirect effect of the C47F polymorphisms on WAPO1 structure, we decide to test this possibility experimentally.

•Line 180: ". homologs" is misspelled.

Author response: In polyploid wheat orthologous genes in the homeologous chromosomes (e. g. 1A, 1B and 1D) are referred to as homeologs (or in the older literature as homoeologs). This is the

© 2024. Published by The Company of Biologists under the terms of the Creative Commons Attribution License (https://creativecommons.org/licenses/by/4.0/).

official nomenclature in wheat and we are required to follow it.

•*Line 211 to 217: The authors describe the expression of FRIZZY PANICLE (FZP) in wheat. I do not understand the rationale for this experiment. Is FZP used as a marker for a specific region of the meristem to better describe the LFY expression profile? I think the authors should better explain their intentions here.*

Author response: The reviewer is correct, *FZP* was used a marker for the initiation of spikelet development in the IM, since *FZP* is not expressed in the IM lateral meristems until the initiation of the terminal spikelet where the first lateral meristems correspond to the glumes of the terminal spikelet. We modified this paragraph making our rationale clear from the first sentence: "We used the gene *FRIZZY PANICLE* (*FZP, TraesCS2A02G116900*) as an early marker for the IM→TS transition." In addition, we added the following sentence to the legend of supplemental figure S4: "*FZP* (TraesCS2A02G116900) is used as a marker for the initiation of spikelet development."

•*Line 278: I think they are referring to Figure 4D and not Figure 5D.*

Author response: The smFISH expression of *WAPO1* is presented in both Fig. 4D (in yellow, to facilitate comparisons with *LFY*) and in Fig. 5D (in blue, to facilitate comparisons with the floral organ identity genes in figure 5D). We added a reference to both figures: "…in a distal region of the developing spikelets that mostly overlapped with the expression of *WAPO1* (Fig. 4D and 5D)."

•*Line 281 to 285: Why did they do the real time at stage W4.0 and not at stage 3.25/3.5 as in the rest of the paper?*

Author response: The previously published results for the expression of the floral organ identity genes in the *wapo1* mutant were performed at W4.0 (Kuzay et al. 2022), so we wanted to use the same developmental stage for the *lfy* mutants to make a valid comparison. Moreover, at W3.25/3.25 the floral organ identity genes are just starting to be expressed in the more mature central spikelets (Fig. 5), and more significant differences between the wildtype and *wapo1* mutant for these genes were observed in the previous study at W4.0 than at W3.25/3.25. We clarified this in the sentence: "Finally, we used qRT- PCR to characterize the effect of the lfy mutation on the expression of the floral organ identity genes in the wheat developing spike at W4.0, when these genes are highly expressed (Kuzay et al., 2022)"

•*Lines 372 to 374: "These results In Arabidopsis." I disagree with this sentence because the interaction between LFY and WAPO1 is not well demonstrated and the data provided by the authors is not sufficient to support this sentence.*

Author response: We eliminated the comparison with Arabidopsis from this sentence and emphasized that *LFY* and *WAPO1* act cooperatively in both the regulation of SNS and the correct formation of loral organs in wheat. We changed the concluding sentence to : "I In summary, *LFY* and *WAPO1* act jointly to regulate both inflorescence architecture and floral organ development in wheat, and likely in other plant species."

•*Line 413 to 414: I think this sentence should be changed to make it clearer to readers.*

Author response: We changed and expanded the sentence to make it clearer to the readers: "In summary, *lfy* mutations have opposite effects on SNS than *vrn1* or *ful2* mutations, and these effects are mostly additive. However, significant genetic interactions between *LFY* and the *SQUAMOSA* genes also contribute to the observed differences in SNS in the different mutant combinations"

•*Line 415: I suggest changing this title.*

Author response: We changed this subtitle to "*LFY* and *WAPO1* show dynamic spatio-temporal expression patterns during wheat spike and spikelet development"

Reviewer 2

© 2024. Published by The Company of Biologists under the terms of the Creative Commons Attribution License (https://creativecommons.org/licenses/by/4.0/).

Comments for the Author

1. The present study by Paraiso et al. applied genetic, biochemical, and molecular approaches to better understand spike development in wheat and its regulation through LFY in combination with WAPO1 and other MADS-box regulators. Even though there are no major concerns related to the gene identification and interaction studies of LFY; it seems difficult for the authors to bring together the detected expression data with the found phenotype—more SNS! Considering the presented work, several issues remain and may require reconsideration or interpretation:

Author response: We agree with the reviewer that it was difficult to reconcile the expression profiles of *WAPO1* and *LFY* with our hypothesis of a difference in the timing of the IM→TS transition between the wildtype and the *lfy* mutant. Since the expression smFISH expression profiles for *LFY* and *WAPO1* were consistent with previously published *in situ* hybridization studies, we performed two additional experiments to re-examine our hypothesis.

The results from the new experiments are described in detail at the beginning of this review (Fig. 3C-D). These experiments show that both *LFY* and *WPAO1* control the rate of formation of the lateral spikelets rather than the timing of the formation of the terminal spikelet. Our assumption that the timing of the IM→TS transition was the main contributor to the differences in SNS was incorrect in the previous discussion.

The two experiments show that the reduced rate of SM formation in both *lfy* and *wapo1* relative to the wildtype are evident from the earliest stages of spike development (Fig. 3C-D). Since at this early stage *WAPO1* and *LFY* are colocalized in the IM, it is now simpler to explain the relationship between the spatio-temporal expression profiles of *LFY* and *WAPO1* and the observed reductions in SNS in the *lfy* and *wapo1* mutants.

LFY is a pioneer transcription factor that can bind nucleosomes in closed chromatin, displace H1 linker histones and recruit the SWI/SNF chromatin-remodeling complex, permitting the binding of other transcription factors (Yamaguchy 2021 Front PL Sci 12:701406). We added this information to the discussion: "LFY is also a pioneer transcription factor that can bind nucleosomes in closed chromatin, displace H1 linker histones and recruit the SWI/SNF chromatin-remodeling complex, permitting the binding of other transcription factors (Jin *et al.*, 2021, Lai *et al.*, 2021, Yamaguchi, 2021)."

Therefore, the overlap of *LFY* and *WAPO1* expression in the IM at the early stage of spike development is likely sufficient to open the chromatin in the promoters of multiple genes facilitating the access of other transcription factors. Once chromatin has been modified to an open state, it can remain open even in the absence of the LFY-WAPO1 complex. Since the timing of the IM→TS transition is not different between the wildtype and both the *lfy* and *wapo1* mutants, it is no longer necessary to have *LFY* and *WAPO1* expression colocalized in the IM at the time of the transition!

In the Discussion section. we replaced previous speculations by the simpler hypothesis that the co expression of *LFY* and *WAPO1* in the IM at the early stages of spike development (Fig. 4A) may be sufficient to activate gene expression networks that accelerate the rate of SM initiation. See proposed Discussion paragraph in the initial discussion presented to the three reviewers.

2. Considerations for LFY: I kind of agree with the authors that wheat LFY affects proper floral development. While this is easy to be explained by the homeotic floret mutant phenotypes (not only in wheat but also from other grasses!), mRNA expression domains in the developing spike and spikelet are less straight forward. If wheat LFY promotes floral development and growth, authors may need to explain why, during DR stage, LFY is only expressed in the leaf ridge (LR) but not in the developing spikelet ridge (SR), and how this might work?

Author response: In wheat, *LFY* does not promote spikelet development, it represses it! This has been also observed in rice and is opposite to what happens in Arabidopsis. This is due to a fundamental difference between these two groups of plants: In Arabidopsis, LFY is required to

© 2024. Published by The Company of Biologists under the terms of the Creative Commons Attribution License (https://creativecommons.org/licenses/by/4.0/).

promote the expression of *AP1* and *CAL*, which determines its major role as a flowering promoter. However, experiments in Arabidopsis have shown that induction of *LFY* with a GR promoter in an *ap1 cal* combined mutant results in a lower proportion of plants with flowers than in the control without *LFY* induction (Goslin et al., 2017). In the grasses, LFY does not control the expression of *VRN1 FUL2 FUL3* (the homologs of *AP1 CAL FUL*) and, therefore, we only see its repressing role, and not a promoting role as in Arabidopsis.

The repressive role of *LFY* in spikelet development is consistent with its exclusion from the early spikelet meristems (Fig. 4B and 4C), a particular spatial distribution that has been also reported in rice. This negative role of *LFY* on spikelet development is also consistent with the drastic reduction of *LFY* hybridization signal relative to the *SQUAMOSA* MADS-box genes from the distal part of the wheat spike between W2.5 and W3.25, at the time of the IM→TS transition.

In the earliest papers, *UFO* was described as a cadastral gene that provides spatial information to the floral organ identity genes. *LFY* was described as a floral meristem identity gene, but that is due to the direct regulation of *AP1* and *CAL* by LFY in Arabidopsis. In the grasses, where *LFY* does not regulate the *SQUAMOSA* genes, both *LFY* and WAPO1 act as cadastral genes providing spatial information to the floral organ identity genes.

3A. One possibility could be that wheat LFY expresses in peripheral domains of the floral meristems and acts non-cell autonomously into the above-lying floral meristem. For example, if wheat LFY mutant (lfy) plants would have a pronounced LR over the SR in the most basal DRs it could be diagnostic for floral promotion in wt plants (i.e. less LR in most basal DRs!). That means lfy plants may have similarly enhanced basal LRs as SVP mutants have (pl. see e.g. Backhaus et al. 2022 and 2023). If confirmed in a small experiment, it may suggest that wheat LFY provides short-range, non-cell autonomous signals for SM formation and growth from the LR. This would be in line with previously proposed peripheral signaling centers occurring in grass inflorescences (pl. see Whipple NP 2017).

Author answer: Since there are no differences in the timing of the IM→TS transition between the wild type and the *lfy* or *wpao1* mutants (see new Figs 1F-G), there is no longer need to postulate a hypothesis on how *LFY* and/or *WAPO1* regulate the transition of the IM to a SM.

We agree with the reviewer that the timing of the IM→TS transition may be regulated by a signal coming from the most developed central spikelets in both the wildtype and *lfy* mutant. We observed a coincidence in the timing of the expression of the floral organ identity genes in the more developed central spikelets and the IM→TS transition. However, this is just a correlation and not a demonstration of causality, so we prefer to avoid speculating about this mechanism. We are currently collaborating with Dr C. Uauy (the senior author of the Backhaus papers) to test this hypothesis.

Wheat has a very simple inflorescence that lacks the complex branching patterns of the panicles, so the complex signaling centers proposed by Whipple (2017) to explain these more complex grass inflorescences are not required here. The reduced areas of *LFY* expression in the distal part of the lateral meristems seem to contribute to the localization of the SM but further research is necessary to establish a causal relationship.

Regarding a possible role of *LFY* in the repression of the lower ridge, we are aware of the paper in Plant Physiology 2022 by Miao et al. suggesting that *APO2=LFY* controls bract development indirectly by the regulation of *OsSPL14/OsSPL17* and *NL1*. However, we could not find evidence of bract outgrowth in the *apo2* mutant. In wheat, we have observed clear de-repression of the lower ridge and bract formation in our *vrn1 ful2* and full leaf restoration in the *vrn1 ful2 ful3* combined mutants (Li et al. 2019). De- repression of the lower ridge has been also described recently in the *spl14 spl17* hexa-mutant in hexaploid wheat (Chen et al. 2023). We also observed a basal bract outgrowth in our unpublished *SPL* mutants, but we have not observed de-repression of the lower ridge in our *lfy* or *wapo1* wheat mutants. Moreover, none of these three genes shows significant differences in expression between the wheat *lfy* and WT at W3.0 (unpublished RNAseq data).

© 2024. Published by The Company of Biologists under the terms of the Creative Commons Attribution License (https://creativecommons.org/licenses/by/4.0/).

3B. The similar idea would be applicable for the later stage expression domains (2.5 and 3.25). Here, wheat LFY expresses in the peripheral zone of the SM in a ring-like band; in an area where already differentiation most likely has occurred towards floret meristem (FM) identity. Unfortunately, authors do not provide data on the later occurring floret related expression domains of wheat LFY. However, from Selva et al 2021 it can be deduced that barley LFY shows FM and floral organ expression patterns, suggesting that wheat LFY may follow a very similar trend. In summary, I thus would encourage authors to at least discuss this above-mentioned possibility during their discussion.

NOTE: Figure provided for reviewer has been removed. It showed Figure 4 from **Shitsukawa, N., Takagishi, A., Ikari, C., Takumi, S. and Murai, K.** (2006). WFL, a wheat FLORICAULA/LEAFY ortholog, is associated with spikelet formation as lateral branch of the inflorescence meristem. *Genes Genet. Syst.* 81, 13-20. doi:10.1266/ggs.81.13

Author answer: We had very limited space for tissues in our spatial transcriptomics study and we prioritized the inclusion of four replications for W2.5 and W3.25 to have sufficient statistical power to compare the expression changes in the distal region during the IM→TS transition. We do not have results for later stages of spikelet development. Fortunately, Shitsukawa et al. (2006) performed excellent *in situ* hybridizations at a later stage when all three florets were developed. These *in situ* experiments clearly show that the band of high *LFY* intensity distal to the lemmas is formed in each floret (see panel B on the right), highlighting its importance for normal spikelet development. We added the following sentence to the discussion: "This intense *LFY* expression band has been also observed distal to the lemma primordia of the second and third florets by in situ hybridization in more developed wheat spikelets (Shitsukawa et al., 2006), highlighting its importance for normal floret development."

4) Considerations for WAPO1 expression domains: I've had a hard time to reconcile the seen expression domains with the proposed function of IM>TS transition and more SNS. Perhaps there's something like protein transport as authors discussed; but less likely. However, an alternative idea could be that WAPO1 is involved in meristem maturation. And if so, high expression of WAPO1 in the SM (W3.25) could produce more SNS through a delayed progression towards FMs in the most matured floral primordia while in parallel expanding the time for TS transition. This might also suggest that WAPO1 and wheat LFY might work collectively during floral progression but in a highly balanced mode modulating SNS. If one of them is lacking, SNS will be low because the other partner "takes-over" and induces premature meristem maturation (wapo1lfy = too low floral induction/promotion). This would also be in line with the double mutant result. Short-range signaling or protein transport from the periphery to the meristem is also in line with the found direct interactions of both proteins.

Author answer: We also had a hard time reconciling the *WAPO1* expression domains with its function in SNS. That is why we performed the additional experiments described at the beginning of this review.

These two experiments showed that both *LFY* and *WAPO1* regulate the rate of SM formation rather than the timing of the IM→TS transition. Therefore, there is no need to postulate long range transport of LFY or WAPO1 proteins, or a signal from the more developed spikelets, since there are no differences in the timing of the IM→TS transition between the mutants and the wildtype.

5) Authors may want to re-assess the ubi-LFY lines and associated phenotypes for the same context. They mentioned here that these lines could only partially complement wt plants and had fertility problems, most likely due to ectopic expression and pleiotropic effects. I assume that the ubi-LFY line also needs to have an up-regulated WAPO1 to facilitate more SNS. A hypothesis to be tested, I guess.

Author response: We intercrossed the UBI:LFY x UBI:WAPO1 but the progeny plants grew poorly and were sterile so we did not characterize them further. Similar experiments combining 35S:UFO and 35S:LFY in Arabidopsis and Petunia resulted in ectopic expression of the floral organ identity genes in the seedling and cotyledons and in arrested growth (Parcy et al. 1998, Nature 395:561 and Souer

© 2024. Published by The Company of Biologists under the terms of the Creative Commons Attribution License (https://creativecommons.org/licenses/by/4.0/).

et al. 2008, The Plant Cell 20:2023).

We added Fig. S2B-C showing floral organ defects and their frequency in the UBI:LFY-HA plants, which explain its higher fertility relative to the *lfy* mutant and its lower fertility relative to the wildtype. See answer 7 to reviewer 1 for the modified text included in the paper.

Reviewer 3

Advance Summary and Potential Significance to Field

The manuscript by Paraiso et al. characterizes lfy mutants in durum wheat, also showing LFY relationship with WAPO1 and some MADS box genes. It also presents a detailed and informative transcriptional profiling of these genes during inflorescence development by smFISH. After screening of a durum wheat mutagenized population, one LFY truncated allele for genome A and one for genome B were isolated and crossed to obtain a lfy mutant. Consistently with LFY role in other species, the mutant showed shorter spikes bringing less spikelets. Interestingly, a dosage effect was also observed with A and B mutants showing intermediate phenotypes. Additionally, lfy mutants showed florets with sovra-numeral organs and mixed identities probably caused by altered expression of MADS box floral identity genes. LFY overexpression under a constitutive promoter could largely revert the lfy mutant defects, confirming LFY as the causal gene. Since LFY interacts with APO1/UFO in other species and similar phenotypes for the mutants were observed, this interaction was also shown in durum wheat by co-IP. smFISH was widely used in wt tissues to study the localization of LFY, WAPO1 and also MADS BOX genes in a temporal manner while expression of some of these genes was also shown deregulated by qRT in lfy mutant smFISH could describe at high-definition and also in a semi quantitative manner the expression dynamics of quite a number of floral identity MADS box genes during development and their dependency on LFY was also suggested. Although at different level, vrn1 and ful2 mutations exacerbated the SNS decrease observed in lfy mutant, showing genetic interaction between these genes, especially for lfy and vrn1. LFY in wheat shows therefore some similarities with Arabidopsis but also many differences, that are shared with other grasses. Overall, the manuscript is very interesting for the field and discusses important similarities between Arabidopsis and grasses and in particular wheat that help understanding molecular pathways that control the development of different types of inflorescences.

Comments for the Author

1) TITLE: I would try to mention the MADS box also in the title, in relation to LFY, as much information on their function is in the manuscript too.

Author answer: The new results show that the rate of SM formation is similarly affected by *lfy* and *wapo1* mutants and we also observed similar floral defects in both mutants. This indicates that, in wheat, there is a large overlap between the function of both genes. Therefore, we expanded the title of the paper to "LEAFY and WAPO1 jointly regulate spikelet number per spike and floret development in wheat"

The interaction between *LFY* and *VRN1* is highly significant but it only explains a small proportion of the total variation in SNS (~20%), so we prefer to keep the title focused on LFY. It is difficult to convey the idea of the relative contribution of the SQUAMOSA genes within the limited number of words allowed in the title. We think is more critical to emphasize the overlap of functions and the physical interaction between LFY and WAPO1.

2) Since the mutation in the B genome is quite at the end of the cds and LFY overexpressing lines in lfy background could not fully complement, other alleles or RNAi would remove any doubt. I understand nevertheless that it might not be easy and especially not fast to do it.

Author answer: The stop codon in LFY-B is at position 249 and results in the elimination of 144 amino acids that represent 36.7% of the protein including the complete DBD. Without a DBD, it is

© 2024. Published by The Company of Biologists under the terms of the Creative Commons Attribution License (https://creativecommons.org/licenses/by/4.0/).

safe to assume that the truncated Kronos lfy-B protein can no longer bind to their normal DNA targets and is no longer functional.

The truncated region includes a very conserved part of the protein as shown in the figure below, and also in the answer to the first question from Reviewer 1. The figure provided in that answer shows conservation of the truncated region among more unrelated taxa, and provides the location of both the *lfy- A* and *lfy-B* selected mutations.

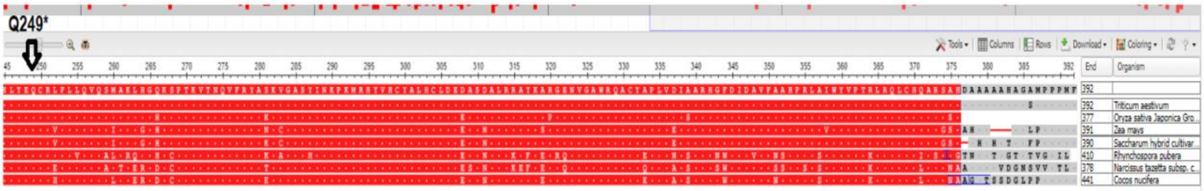

We added this information in the description of the mutants in the first section of results: "The eliminated amino acids in the two wheat mutants include the highly conserved LFY DNA binding domain, suggesting that the truncated proteins can no longer bind their target DNAs and are, most likely, not functional (Maizel *et al*., 2005, Rieu *et al*., 2023b) (Fig. 1A)."

The strong phenotypes of the combined *lfy-A lfy-B* mutations, and their almost identical effect on SNS to the *wapo1* CRISPR mutants also suggest that the encoded truncated proteins are no longer functional (please see more detailed answer to reviewer 1 question 1 above).

3) Concerning LFY/WAPO interaction:-smFISH show very nicely where molecules of LFY or WAPO1 mRNA are found: although it is proposed that the two proteins work together, I am not sure that their expression largely overlaps. It looks that only few cells show both transcripts, and only in specific developmental stages. Do you think that the proteins might move?

Author answer: We included a new supplemental Fig. S6 showing in more detail the overlap between the expression profiles of *LFY* and *WAPO1* in four spikelet meristems at the lemma primordia (LP) stage (see also answer to Reviewer 1 question 6 above). This new smFISH picture show that *LFY* and *WAPO1* are co-expressed in multiple cells in the spikelet/floral meristem region, and therefore there is no need to postulate additional movement of their encoded proteins. Moreover, the floral organ identity genes, which are similarly regulated by both *LFY* and *WAPO1*, are also expressed in the *LFY-WAPO1* overlapping region, providing additional indirect evidence that the two genes are colocalized in this region.

-Although WAPO-LFY interaction was shown in other species and it was not surprising, in wheat too it could be confirmed by another method other than co-IP. Also: how is the complex formed? Is it a dimer or a tetramer? Could you conclude something on that? LFY-WAPO interaction was tested using two allelic variants of WAPO but which is Kronos's variant?

Author answer: As requested by the reviewer, the first time we mention the *WAPO1* alleles we clarified that in Kronos WAPO1 carries the 47C allele: "We used co-immunoprecipitation (Co-IP) to test the interaction between LFY-A and two WAPO-A1 natural alleles that differ in the presence of a cysteine or a phenylalanine at position 47 to test if this polymorphism affects the interaction (Kronos carries the 47C allele)."

Regarding the validation of the coIP results by other methods, we tried Y2H but did not see an interaction in yeast. We think that a coIP interaction in wheat cells is more relevant to this study than an interaction in yeast. Moreover, this interaction has been demonstrated *in planta* by multiple methods in a large number of species including several grasses, so we considered the clear coIP experiment sufficient as a confirmatory result.

4) Which wapo1 mutants were used in this experiment? Which are the alleles? (please specify in

© 2024. Published by The Company of Biologists under the terms of the Creative Commons Attribution License (https://creativecommons.org/licenses/by/4.0/).

line 175).

Author answer: We added the requested information in the paragraph suggested by the reviewer: "To test if the physical interaction between LFY and WAPO1 was reflected in a genetic interaction for SNS, we intercrossed *lfy* with a loss-of-function *wapo1* mutant containing early truncation mutations in both *WAPO-A1* and *WAPO-B1* (Kuzay et al., 2022)."

5) how do wapo1 florets look like? Are they similar to lfy? Is there an additive effect between the two mutations more prominent that for SNS, that, although significant, is very low?

Author answer: The floral defects in *wapo1* are almost identical to those in *lfy*. Both mutants show frequent fusions of lodicules with other organs, reduced number of stamens and fusions, multiple carpels, and conversions of organs to membranous tissues. We added a brief description of the previously published *wapo1* mutants and directed the readers to the Kuzay et al. (2022) reference. Below is a side- by-side comparison of the picture from this and the previous *wapo1* paper. We did not characterize the combined *lfy wapo1* mutant for floral defects since it was not the main focus of this paper.

NOTE: Figure provided for reviewer has been removed. It showed Figure 3 A-D from **Kuzay, S., Lin, H., Li, C., Chen, S., Woods, D., Zhang, J., and Dubcovsky, J.** (2022). WAPO-A1 is the causal gene of the 7AL QTL for spikelet number per spike in wheat. *PLoS Genet.* 18: e1009747.

6) Concerning VRN1 and FUL it seems that their expression also only partially overlaps with LFY and WAPO, this also implies some protein movement or some cell specificity of MADS activation by LFY/WAPO

Author answer: *VRN1* and *FUL2* are both expressed throughout the developing spikes so they overlap with both *LFY* and *WAPO1* expression domains. Therefore, there is no need to postulate movement of the encoded proteins. Moreover, we do not claim a direct protein interaction between LFY and the SQUAMOSA proteins. We think that the genetic interaction between *LFY* and the *SQUAMOSA* genes is the result of the overlap among the target genes they regulate. In Arabidopsis, it has been demonstrated that there is a substantial overlap among the direct targets of LFY and AP1, and that some of those targets are regulated in opposite directions. We have not published it yet, but our RNAseq results comparing the differentially expressed genes (DEGs) between *lfy* and Kronos wildtype with the DEGs between Kronos wildtype and the *vrn1 ful2* combined mutant showed an overlap in 126 genes, 99 of which are regulated in opposite directions. We think that these common targets may contribute to the significant genetic interaction between *LFY* and *VRN1*.

7) I would move the model in fig S11 (maybe simplified) to the main text…

Author answer: This is still a preliminary working model that is not prominently mentioned in the text, so our preference is to keep it in the supplementary materials. In addition, there are limitations of space and we already have six large figures in the main text.

8) LINE 189 please specify what variety is CS

Author answer: We added: "A previous RNA-seq study including different tissues at different developmental stages in Chinese Spring (CS), …"

9) LINE 211-217: I do not see the point of adding here FZP, it is not introduced, and it is not useful to understand LFY's function in my opinion.

Author answer: *FZP* is just a marker for the initiation of spikelet development. The comparison between the IM before and after the transition to a terminal spikelet requires an early marker of

© 2024. Published by The Company of Biologists under the terms of the Creative Commons Attribution License (https://creativecommons.org/licenses/by/4.0/).

spikelet development. The presence of FZP at the axils of the future glumes is an important marker for the transition of the lateral meristems into glumes rather than spikelets. This marker was useful to select sections where the IM was transitioning to a terminal spikelet (W3.25) from younger stages where the IM was still generating lateral SM (W2.5), for our *SQUAMOSA*/*LFY* studies. We clarified in the text that *FZP* was used as a marker: "We used the gene *FRIZZY PANICLE* (*FZP*, *TraesCS2A02G116900*) as an early marker for the IM→TS transition." In addition, we added the following sentence to the legend of supplemental figure S4: "*FZP* (TraesCS2A02G116900) is used as a marker for the initiation of spikelet development."

10) Why in figure 1C the experiment was done on 9 plants while in FigS1 on 27? Are they two independent experiments?

Author answer: Yes, there are two different experiments. The experiment in 1C is to test the large differences in SNS between the WT and the *lfy* mutant, so 9 plants are sufficient to provide adequate statistical power. However, the experiment for Fig. S1 also includes the individual homeologs, which show more subtle differences. These smaller differences in SNS require a larger number of plants to obtain a similar statistical power. In addition, the experiment in Fig.S1 was designed to test the interaction between *lfy-A* and *lfy-B*. Interaction effects are usually smaller than the main effects and require a larger number of replications to generate adequate statistical power.

Second decision letter

MS ID#: DEVELOP/2024/202803

MS TITLE: LEAFY and WAPO1 jointly regulate spikelet number per spike and floret development in wheat

AUTHORS: Francine Paraiso, Huiqiong Lin, Chenxia Li, Daniel P. Woods, Tianyu Lan, Connor Tumelty, Juan M Debernardi, Anna Joe, and Jorge Dubcovsky
ARTICLE TYPE: Research Article

I am happy to tell you that your manuscript has been accepted for publication in Development, pending our standard publication integrity checks. The reviewers appreciate your efforts to address their concerns. Reviewer 3 has a remaining question that would be nice to address if you are able in the final text.

Reviewer 1

Advance summary and potential significance to field

In the manuscript "LEAFY regulates spikelet number per spike and floret development in wheat", the authors describe the role of LEAFY in spikelet development in wheat. To investigate the role of LEAFY in this process, they identified mutants for LFY-A and LFY-B and analysed the effect of this mutation on spikelet number per spike (SNS) and flowering time. They found that lfy shows less SNS compared to the wild type, but this phenotype is rescued when LFY-A is overexpressed in lfy under the Ubiquitin promoter. They then investigated the molecular mechanism by which LFY controls spikelet number. To do this, they first suggested that LFY function is mediated by its interaction with WAPO1 by studying their interaction by co-immunoprecipitation, the lfy wapo1 double mutant phenotype and their expression localisation in wheat spikelets at different time points during development. They also studied the spatial localisation of several VRN1 and FUL2, which are SQUAMOSA MADS box transcription factors involved in spikelet development and the IM>TS transition. They observed that the expression of VRN1 and FUL2 in lfy is comparable to that of the wild type and they characterised their spatial localisation in the wild type. In parallel, they also studied the distribution of floral identity genes (AP3, PI1, AG1, AG2, SEP1-2, SEP1-4, SEP1-6, SEP3-1

© 2024. Published by The Company of Biologists under the terms of the Creative Commons Attribution License (https://creativecommons.org/licenses/by/4.0/).

and SEP3-2) in the wild type and described their colocalisation with LFY at W3.25 stage. Finally, they studied the genetic interaction between LFY and VRN1 and FUL2 and their effect on SNS. Nevertheless, I think that in the future it will be challenging and interesting to understand the role of the few cells that show co-expression and probably LFY-WAPO1 interaction and to compare their role with that of the cells in which these two proteins do not colocalise.

Comments for the author

After the extensive revision by the authors, I find that they have addressed all the doubts I had about this manuscript with convincing arguments. I thank the authors for taking my comments seriously and addressing them in a professional manner.

Reviewer 2

Advance summary and potential significance to field

Please see my previous review

Comments for the author

After seeing and reading the revised version of this manuscript I can clearly realize the efforts authors have put into this, in particular by reconsidering previous ideas! The message appears much more nuanced and toned-down. The new direction of data interpretation shifted from the obscure TS->IM transition to the rate of SM and floret maturation, which, from my point of view, is a simpler and more solid working hypothesis. Moreover, by providing new experiments authors solidified this point! Importantly, by swapping previously embosomed ideas authors showed great attitude and by doing this may provide a more accessible baseline for future work related to these proteins. I am very delighted to realize that authors considered many of my points as being important and valid. I very much appreciate the efforts made; and thus, I am very much looking forward to seeing this work being published.

Reviewer 3

Advance summary and potential significance to field

dear Author,
thank you.
I am fully satisfied by the revisions and I think that this work adds significant understanding to the role of LFY and APO in flower development in cereals.

Comments for the author

I have still one minor concern, that perhaps arises from my not deep knowledge of wheat inflorescence development, on the new figure 3C + D: if LFY and WAPO are expressed since early stages on IM development, why do you actually see a difference in SM number only after about 28d?

© 2024. Published by The Company of Biologists under the terms of the Creative Commons Attribution License (https://creativecommons.org/licenses/by/4.0/).
